# Lactylation of Mutation-Prone RAS Residues Mimics Oncogenic Mutations and Drives Constitutive Activation

**DOI:** 10.34133/cancomm.0047

**Published:** 2026-08-26

**Authors:** Ruocen Liao, Chenglong Ma, Xingyu Chen, Longchang Bai, Qianhua Cao, Yu Zhang, Chenfang Dong

**Affiliations:** ^1^ Department of Surgical Oncology (Breast Center) and Department of Pathology and Pathophysiology, Key Laboratory of Cancer Prevention and Intervention, Ministry of Education, The Second Affiliated Hospital, Zhejiang University School of Medicine, Hangzhou, Zhejiang, P. R. China.; ^2^ Department of Breast Surgery, First Affiliated Hospital, Zhejiang University School of Medicine, Hangzhou, Zhejiang, P. R. China.; ^3^ Department of Respiratory and Critical Care Medicine, Key Laboratory of Respiratory Disease of Ningbo, The First Affiliated Hospital of Ningbo University, Ningbo, Zhejiang, P. R. China.

## Abstract

**Background:** Elevated expression of wild-type RAS contributes to tumorigenesis, yet the mechanisms underlying its mutation-independent hyperactivity remain unclear. Lactylation, a lactate-derived post-translational modification, has emerged as a regulator in cancer, but its role in RAS activation is unknown. Here, we investigated whether lactylation of RAS at specific residues drives its constitutive activation and the underlying mechanisms. **Methods:** Lactylation sites on RAS were identified by mass spectrometry. A site-specific lactylation system was employed to introduce lactyl-lysine into RAS at K117 and K147. Structural effects were analyzed via molecular dynamics simulations. RAS activity was assessed through GTP-binding and GTPase assays; protein stability was evaluated using ubiquitination mutants and cycloheximide chase. The responsible enzymes were defined by in vitro lactylation and delactylation assays. Functional impact was tested via colony formation, mammosphere assays, and xenograft models. Clinical relevance was examined in breast cancer tissues and survival databases, and drug synergy was assessed by combining lactate-lowering agents with MEK inhibitors. **Results:** We reported that RAS was lactylated at K117 and K147, 2 residues mutated in cancers. Mechanistically, RAS lactylation was catalyzed by TIP60 and reversed by SIRT2. Lactylation at these residues recapitulated features of oncogenic mutations by disrupting GTP interaction, impairing intrinsic guanosine triphosphatase (GTPase) activity, and competitively antagonizing K48-linked ubiquitination at K147 to stabilize RAS. Functionally, RAS lactylation promoted malignant transformation and tumorigenesis, phenocopying oncogenic mutations at the same sites. Inhibition of lactate production sensitized lactylated RAS-driven cancer cells to drugs targeting RAS mutations, revealing a therapeutic vulnerability. Clinically, RAS lactylation was elevated in breast cancer tissues and correlated with poor prognosis. Employing a site-specific lactylation system, we further confirmed that lactylation alone phenocopied mutation-driven RAS activation. **Conclusions:** Our study redefines lactylation as a functional “mutation mimic” mechanism, which not only recapitulates the effects of oncogenic mutations but also bridges the gap between genetic and epigenetic drivers in cancer. This suggests that targeting the lactylation pathway alone or in combination with mutation-directed therapies represents a promising strategy for treating wild-type RAS-driven cancers.

## Background

The small GTPase RAS (KRAS, HRAS, and NRAS) functions as a molecular switch, cycling between an inactive guanosine diphosphate (GDP)-bound state and an active guanosine triphosphate (GTP)-bound state [1]. This switch is governed by a conserved guanine nucleotide-binding (G) domain that has 5 conserved guanine nucleotide-binding boxes (G boxes) [1]. In the normal resting state, RAS is primarily GDP-bound and inactive due to the intrinsic GTPase activity of RAS that hydrolyses GTP to GDP. Upon extracellular stimulation of receptor tyrosine kinases (RTKs) or other receptors, RAS-GTP is rapidly and transiently formed, thereby activating diverse downstream signaling pathways, such as the RAF–mitogen-activated protein kinase kinase (MEK)–extracellular signal-regulated kinase (ERK) and phosphoinositide 3-kinase (PI3K)–Akt–mTOR (mammalian target of rapamycin) pathways [2]. *RAS* genes are the most frequently mutated oncogene family, with mutations occurring in nearly 20% of all cancers [3]. Oncogenic mutations in *RAS*, most frequently at glycine 12/13 (G12/G13), glutamine 61 (Q61), lysine 117 (K117), and lysine 147 (K147), lock the protein in a constitutively active GTP-bound state to drive uncontrolled proliferation and tumorigenesis [4].

Wild-type (WT) RAS plays critical roles in cancers in a context-dependent manner [5]. WT-RAS is hyperactivated in numerous cancers lacking canonical mutations. For example, approximately 30% of neuroendocrine prostate tumors exhibit elevated RAS activity without mutations [6]. Some studies have shown that RAS is frequently activated in breast cancer, especially basal breast cancer that lacks mutant RAS, and that this activation confers a mesenchymal phenotype to breast cancer cells [7]. Furthermore, WT-RAS overexpression alone suffices to transform normal fibroblasts [8]. These observations underscore the existence of nonmutational mechanisms capable of breaching RAS activation thresholds. However, the molecular basis for WT-RAS hyperactivity remains poorly understood, representing a critical gap in our understanding of RAS-driven oncogenesis.

A key clue lies in the Warburg effect, a metabolic hallmark of cancer characterized by excessive lactate production [9]. Beyond its role as a metabolic byproduct, lactate serves as a substrate for lysine lactylation, a post-translational modification (PTM) linked to human diseases such as inflammation and cancer [10–15]. However, whether lactylation of RAS occurs and contributes to its activation remains unknown. In this study, we aimed to investigate whether RAS is lactylated at specific residues and to elucidate the functional consequences and regulatory mechanisms of this modification in tumorigenesis.

## Methods

### Plasmids and antibodies

Human *HRAS*, *KRAS*, *AARS1*, *CBP*, *TIP60*, *p300*, *PCAF*, *GCN5*, *SIRT1-5*, *LZTR1*, *USP32*, and *LDHA* genes were amplified from MDA-MB-231 cDNA library and subcloned into pLVX-Puro vector (VL3001, Inovogen) or pcDNA3.1 vector (V79020, Thermo Fisher) for lentiviral or transient expression, respectively. Antibodies specifically recognizing lactylation at lysines 117 and 147 were prepared commercially by immunizing rabbits at Hangzhou Tuoshi Biotechnology Co. Ltd. All other plasmids and antibodies used in this study are specified in the corresponding experimental sections below.

### Cell culture

All cells used in this study were obtained from the American Type Culture Collection (ATCC), where the cell lines were authenticated by short tandem repeat (STR) profiling prior to distribution. The mouse embryonic fibroblast cell line NIH/3T3, human breast ductal carcinoma cell line Hs578T, human triple-negative breast cancer cell line MDA-MB-231, human breast cancer cell line SUM159, and human embryonic kidney cell line HEK293T cells were grown in Dulbecco’s modified Eagle’s medium (DMEM; 11965-092, Gibco) with 10% fetal bovine serum (FBS) (A5256701, Thermo Fisher). The human breast cancer cell line MDA-MB-468 was cultured in Leibovitz’s L-15 medium (11415064, Thermo Fisher ) with 10% FBS (A5256701, Thermo Fisher). All the cells were cultured and stored according to the instructions from the ATCC. For establishing stable transfectants with overexpression of WT- or mutated HRAS, stable clones were selected using 300 ng/ml puromycin (A1113803, Thermo Fisher) for 4 weeks.

### Intracellular expression of site-specific lactylated proteins

To achieve site-specific incorporation of lactyl-lysine (Kla) into RAS at K117/K147, we engineered a eukaryotic orthogonal system combining chimeric ribozymes derived from *Methanocaldococcus jannaschii* and *Pyrococcus horikoshii* transfer RNA (tRNA) synthetases to enhance amber codon suppression efficiency. Four tandem copies of the orthogonal tRNA sequence were cloned downstream of a U6 promoter to improve tRNA–ribosome binding. HEK293T cells were cotransfected with a pcDNA3.1 vector encoding HRAS/KRAS containing the HRAS-K117TAG or HRAS-K147TAG (V79020, Thermo Fisher) and the ribozyme–tRNA plasmid (105830, Addgene) at a 3:1 ratio (μg/μg). After 12 h, the medium was replaced with DMEM supplemented with 2 mM Kla (synthesized by GL Biochem, Shanghai, China) and 10% FBS (A5256701, Thermo Fisher), followed by 48-h incubation. The intracellularly expressed lactylated proteins were confirmed with site-specific antibodies (custom-generated; see the Plasmids and antibodies section) and further analyzed. This system minimizes misincorporation and truncation artifacts by leveraging ribozyme-enhanced tRNA charging and stoichiometric plasmid tuning, distinguishing it from conventional genetic code expansion approaches that rely solely on orthogonal synthetases. The protocol’s reproducibility was validated across 3 independent experiments.

Due to the inherent challenges of amber suppression, such as the reduced efficiency of orthogonal tRNA/aminoacyl-tRNA synthetase pairs, genetic code expansion systems that utilize an amber stop codon (UAG) to incorporate Kla often result in less efficient expression of modified proteins than WT-RAS. To ensure a balanced comparison, the expression of K117/K147 mutant constructs was adjusted to match that of HRAS-WT through optimization of transfection parameters. Specifically, we reduced the amount of WT-RAS plasmid to reduce its expression level, adjusting the ratio of WT-RAS to RAS plasmid containing an amber mutation to 1:4. Additionally, we supplemented with higher levels of Kla (5 mM; synthesized by GL Biochem, Shanghai, China ) to improve the incorporation efficiency of lactyl-lysine at the UAG codon. These optimizations allowed us to achieve similar expression levels of K117/147 lactylated RAS and WT-RAS.

### Mass spectrometry targeting HRAS lactylation and ubiquitination

For comprehensive analysis of HRAS lactylation and ubiquitination, HEK293T cells stably expressing Flag-tagged HRAS were lysed in ice-cold radioimmunoprecipitation assay (RIPA) buffer [50 mM tris–HCl pH 7.4, 150 mM NaCl, 1% NP-40, 0.1% sodium dodecyl sulfate (SDS); 9806, Cell Signaling Technology (CST)] supplemented with protease inhibitors (Roche) and 20 mM N-ethylmaleimide (NEM; 23030, Thermo Fisher) to preserve ubiquitination. Lysates were clarified by centrifugation (15,000*g*, 15 min, 4 °C), and protein concentrations were quantified using a bicinchoninic acid (BCA) assay (Beyotime Biotech). Equal amounts of protein were incubated with anti-Flag M2 affinity gel (Sigma-Aldrich) overnight at 4 °C with gentle rotation. Beads were washed 6 times with lysis buffer, and bound proteins were eluted using 3× Flag peptide (150 ng/μl, Sigma-Aldrich). Eluates were subjected to in-solution tryptic digestion (1:50 enzyme-to-protein ratio, 37 °C, 16 h) or immunoprecipitation with a pan-lysine lactylation (Kla) antibody (PTM-1404, PTM Bio; 4 °C, overnight) to enrich lactylated peptides.

For ubiquitination analysis, tryptic peptides were desalted using C18 StageTips (Thermo Scientific), while Kla-enriched peptides were vacuum-dried and reconstituted in 0.1% formic acid. Liquid chromatography–tandem mass spectrometry (LC-MS/MS) was performed on a Q Exactive HF-X system (Thermo Scientific) coupled to an EASY-nLC 1200 ultrahigh-performance liquid chromatography (UHPLC). Peptides were separated on a 75 μm × 25 cm C18 column (1.9 μm beads, Dr. Maisch) using a 90-min gradient of 5% to 35% acetonitrile in 0.1% formic acid at 300 nl/min. MS data were acquired in data-dependent acquisition (DDA) mode with a full-scan range of 350 to 1,500 mass/charge ratio (*m*/*z*) (resolution, 60,000) and top 20 MS/MS scans [resolution, 15,000; higher-energy collisional dissociation (HCD) collision energy, 28%].

Raw files were processed using MaxQuant (v2.4.9.0) against the UniProt human database (20,430 entries) with carbamidomethylation (C) as a fixed modification, and lactylation (K), ubiquitination (K), oxidation (M), and acetylation (N-terminal) as variable modifications. Trypsin/P specificity was set with up to 2 missed cleavages. Mass tolerances were 20 ppm (precursor) and 0.04 Da (fragment). False discovery rates (FDRs) were controlled at <1% using a target-decoy approach. Site localization probabilities were calculated using the PTM scoring algorithm in MaxQuant, with lactylation/ubiquitination sites retained only if localized with ≥75% confidence. Data were further validated using pFind Studio (v3.1.6) with identical parameters to ensure reproducibility. This dual-search strategy minimized false positives and reinforced site-specific modification assignments.

### Molecular dynamics simulations

Molecular dynamics (MD) simulations were conducted to investigate structural and dynamic perturbations induced by lactylation-mimicking mutations (K117Q, K117R, K117N, K147Q, K147R, and K147N) in HRAS. Initial structures of WT and mutant HRAS were generated using AlphaFold 3 with default parameters, followed by energy minimization in implicit solvent to resolve steric clashes. All simulations were performed using BIOVIA Discovery Studio (DS) with the CHARMM36m force field. MD simulations were performed using the standard dynamics cascade protocol in 4 steps: (a) minimization; (b) heating and cooling; (c) equilibrium run; (d) production run for all HRAS and mutants as previously reported [16]. Root mean square deviation (RMSD) of Cα atoms and GTP-binding pocket residues, along with root mean square fluctuation (RMSF) of Cα atoms, were calculated to assess structural stability and flexibility. Hydrogen-bond occupancies between GTP and key residues (e.g., K117, K147, and Q61) were quantified using a geometric criterion (donor–acceptor distance < 3.5 Å, angle > 120°). Visualization and trajectory analysis utilized UCSF Chimera and DS Visualizer.

### Colony formation assay

The colony formation assay was performed using a dual-layer soft-agar system to assess malignant transformation. Briefly, a base layer of 0.7% agarose (Sigma-Aldrich) in complete DMEM (10% FBS, 1% penicillin–streptomycin) was solidified in 24-well plates. A top layer of 0.35% agarose containing 5,000 cells/well (NIH/3T3 or tumor cells) in the same medium was overlaid. Plates were incubated at 37 °C in 5% CO₂ with weekly replenishment of 100 μl of fresh medium to prevent desiccation. After 21 to 28 d, colonies were stained with 0.005% crystal violet (Sigma-Aldrich) in phosphate-buffered saline (PBS) for 2 h, washed, and air-dried. Colonies larger than 50 μm in diameter (visible without magnification) were counted using an automated colony counter (GelCount, Oxford Optronix) with threshold settings optimized to exclude debris. Triplicate wells per condition were analyzed, and data were normalized to control groups. This protocol, optimized for RAS-transformed cells, ensures reproducibility by standardizing agar density, cell seeding, and quantification criteria, distinguishing it from prior methods focused on suspension cultures or nonquantitative imaging.

### GTPase assay

HEK293T cells expressing Flag-tagged HRAS-WT, HRAS with site-specific lactylation (treatment with 5 mM lactyl-lysine), and HRAS K117Q were lysed and immunoprecipitated with anti-Flag M2 affinity gel (Sigma-Aldrich). The bound proteins were eluted and purified for GTPase assay. Intrinsic GTPase activity for WT-, lactylated, and mutant HRAS proteins was measured by the Phosphate Assay Kit (Sigma-Aldrich) to continuously measure phosphate release following the manufacturer’s recommended protocol. Briefly, 25 μl of a 1:10 dilution was used to determine the inorganic phosphate concentration, 50 μl of the reagent was added to the sample to be measured, and, after 30 min in the dark, OD 620 was measured in a spectrophotometer (Thermo Fisher). Each 96-well plate contains a calibration curve for the assessment of phosphate concentration.

### Nucleotide exchange analysis

HRAS protein was saturated with GDP and brought to a concentration of 1 mg/ml in a buffer containing 25 mM tris and 150 mM NaCl. The protein was then used at a final concentration of 700 nM and combined with 1.5 mM mant-GTP (prepared in 25 mM tris, 75 mM NaCl, 5 mM MgCl₂, 25 mM EDTA) in a 96-well plate. Fluorescence intensity was monitored at 20-s intervals for approximately 17 min using a Synergy H1 microplate reader (BioTek), with excitation and emission wavelengths set at 350 and 440 nm, respectively. Data were collected and processed with GraphPad Prism (GraphPad Software Inc.). All experiments were conducted with 3 technical replicates.

### GTP-sepharose binding assay

The GTP-binding assay was performed on HEK293T cells with overexpressed HRAS proteins. The cells were lysed in a buffer (50 mM tris–HCl pH 7.4, 150 mM NaCl, 1 mM EDTA, 1% Triton X-100) supplemented with protease inhibitors (Roche Applied Science) with rotation for 1 h at 4 °C. After centrifugation of the lysates at 21,000*g* and 4 °C for 10 min, equal protein amounts in the supernatants were incubated with 30 μl of GTP-agarose (Sigma-Aldrich, G9768) with rotation at 4 °C for 4 h. The GTP-agarose beads were then washed 3 times with lysis buffer, eluted using 4× loading buffer, and finally analyzed via Western blotting.

### Expression and purification of GST-proteins and RAS activity assay

Chemically competent *Escherichia coli* BL21-AI were transformed with glutathione *S*-transferase (GST)-protein plasmids, cultured in 2× YT medium with ampicillin, and induced with 0.2% arabinose and 1 mM isopropyl-β-d-thiogalactopyranoside (IPTG) at 16 °C for 24 h. Cells were harvested, resuspended in lysis buffer [50 mM tris pH 7.5, 500 mM NaCl, 0.1 mM EDTA, 1 mM dithiothreitol (DTT), 0.1 mM phenylmethylsulfonyl fluoride (PMSF), 0.1 mg/ml deoxyribonuclease (DNase) I, protease inhibitor], sonicated, and centrifuged. Cleared lysates were incubated with Streptactin Beads 4FF, washed, and eluted with 2.5 mM desthiobiotin. Eluted proteins were concentrated and purified via size exclusion chromatography (SEC) (Sephacryl S-200), and protein concentration was determined by A280 absorption.

Cells were grown to 50% to 70% confluence for 3 d, washed with ice-cold PBS, and lysed in lysis/wash buffer (MLB; Millipore), and 500 μg of protein lysate was adjusted to 500 μl with MLB. Active RAS was coimmunoprecipitated using Flag-M2 agarose or specifically pulled down with GST fusion protein of the purified Raf1-RAS binding domain (RBD) and glutathione agarose resin. Bound HRAS was detected by electrophoresis and immunoblotting.

### In vitro lactylation assay

The GST-tagged TIP60 proteins (K314-380G, Sinobiological) were incubated with Flag-tagged HRAS proteins, purified from HEK293T cells, in reaction buffer (50 mM Hepes pH 7.5, 30 mM KCl, 0.25 mM EDTA, 5 mM MgCl_2_, and 2 mM DTT) containing 20 mM lactyl-CoA (coenzyme A). Reactions were carried out at 37 °C for 1 h, followed by the addition of 5× SDS loading buffer and boiling at 100 °C for 5 min. Samples were resolved by sodium dodecyl sulfate–polyacrylamide (SDS-PAGE) and subjected to immunoblotting using specified antibodies to detect lactylation modifications.

### In vitro delactylation assay

An in vitro delactylation assay was conducted using His-tagged SIRT2 (S36-30H) and Flag-tagged HRAS K117La/K147La proteins purified from HEK293T cells. The reaction mixture included 50 mM Hepes (pH 7.5), 1 mM EDTA, 5 mM MgCl₂, 2 mM DTT, and 1 mM NAD^+^ [nicotinamide adenine dinucleotide (oxidized form)]. After incubation at 37 °C for 1 h, 5× SDS loading buffer was added, followed by boiling at 100 °C for 5 min. Samples were then subjected to SDS-PAGE and immunoblotted with relevant antibodies.

### Immunoprecipitation and Western blotting

Cells were lysed in 0.3% Nonidet P40 buffer (50 mM tris–HCl, 150 mM NaCl, pH 7.5) containing inhibitors (1 μg/ml of aprotinin, 1 mM PMSF, 1 μg/ml of pepstatin, 1 μg/ml of leupeptin, 1 mM Na_3_VO_4_, 30 μM trichostatin A (TSA), 1 mM NaF, and 15 mM nicotinamide all at final concentrations). Cell debris was removed by centrifugation at 12,000*g* for 15 min at 4 °C, and the lysate was incubated with agarose conjugated to the corresponding antibodies for 3 h at 4 °C. Immunoprecipitates were washed 3 times with 0.3% Nonidet P40 buffer, then boiled and analyzed by Western blotting. For Western blotting, protein concentration was measured using the Bradford assay kit (Fude Biological Technology). Adjusted protein samples were mixed with loading buffer and electrophoresed on 12% SDS-PAGE gels. After electrophoresis, proteins were transferred to polyvinylidene fluoride (PVDF) membranes (BSP0161, PALL). The membranes were blocked with 5% fat-free milk for 1 h at room temperature. Blocked membranes were incubated with indicated diluted primary antibody following manufacturer’s recommendations and gentle shaking at 4 °C overnight. The primary antibodies used included those against RAS (ab108602, Abcam), HRAS (ab32417, Abcam), KRAS (12063-1-AP, Proteintech), NRAS (CY5418, Abways), FLAG (F3165, Sigma-Aldrich), hemagglutinin (HA) (ab236632, Abcam), β-actin (AC038, ABclonal), ERK (M5670, Sigma-Aldrich), phospho-ERK (9102, CST), MEK1/2 (8727, CST), phospho-MEK (9154, CST), pan-Kla (PTM-1401RM, PTMBio), green fluorescent protein (GFP) (sc-9996, Santa Cruz), and GST (2625, CST). Site-specific lactylation was detected using custom-generated K117La and K147La antibodies (see the Plasmids and antibodies section). The blots were visualized using the enhanced chemiluminescence assay after incubation with the secondary antibody on the next day.

K117 and K147 are located on the G4 box and G5 box, which are highly conserved in the RAS family and even its superfamily (over 150 members). To avoid cross-recognition caused by highly conserved or even identical sequences among RAS family members, for RAS lactylation detection, we used RAS antibodies to coimmunoprecipitate endogenous RAS from tumor cells or tissues, and then performed Western blot analysis with RAS lactylation-specific antibodies.

### Peptide synthesis for immunization of rabbits and dot blot assays

Peptides for immunization of rabbits and dot blot assays were synthesized at Tufts Medical School on a 0.1 mmol scale. The sequences were as follows: HRAS-K117-1, CEKKVLVGNKSD; HRAS-K117La-1, CEKKVLVGNK(La)SD; HRAS-K117-2, GNKSDLPSRTEEKC; HRAS-K117La-2, GNK(La)SDLPSRTEEKC; HRAS-K147-1, CKEEIETSAKTRQ; HRAS-K147La-1, CKEEIETSAK(La)TRQ; HRAS-K147-2, TSAKTRQREEKC; HRAS-K147La-2, TSAK(La)TRQREEKC. Peptides were diluted to 1 μM for assays. For dot blots, 1 μl of peptide solution was spotted onto a nitrocellulose membrane, forming 3- to 4-mm spots. After drying, membranes were blocked with tris-buffered saline with 0.1% Tween 20 (TBST) containing 5% nonfat milk, followed by immunoblot analysis as described [17].

### Mammosphere assay

Mammosphere assays were performed following the protocol previously described [18]. Briefly, mammosphere formation was assessed by seeding single-cell suspensions (2,000 cells/ml) in ultra-low attachment 6-well plates (Corning) with serum-free mammosphere medium [DMEM/F12 supplemented with 20 ng/ml epidermal growth factor (EGF), 10 ng/ml basic fibroblast growth factor (bFGF), 10 ng/ml B27, 4 μg/ml heparin, and 1% penicillin–streptomycin]. Plates were incubated at 37 °C under 5% CO₂ for 10 to 14 d, with 50% medium replacement every 3 d. Spheres >50 μm in diameter were counted using a Celigo Image Cytometer (Nexcelom) after staining with 0.1% trypan blue to exclude debris. Data were normalized to the initial seeding density and expressed as sphere-forming efficiency (SFE). This protocol, optimized for RAS-transformed cells, includes heparin to inhibit cell aggregation, distinguishing it from prior methods focused on primary mammary stem cells.

### Tumorigenesis assay

Animal experiments were performed according to procedures approved by the Institutional Animal Care and Use Committee at Zhejiang University (approval number: AIRB-2025-0233). Female BALB/c nude mice (5 to 6 weeks old) were purchased from Shanghai Model Organisms Center Inc. and maintained under specific pathogen-free conditions at 22 ± 2 °C with a 12-h light/dark cycle, with free access to food and water. To evaluate the tumorigenic potential of lactylated RAS, female BALB/c nude mice (5 to 6 weeks old) were randomized into groups (*n* = 6 per group) and injected subcutaneously in both flanks with 1 × 10^6^ cells stably expressing HRAS-WT, lactylation-mimicking mutants (K117Q/K147Q), or vector control suspended in 100 μl of PBS:Matrigel (1:1). Mice were monitored twice weekly for tumor formation using digital calipers. Tumor volume was calculated as (length × width^2^)/2. Mice were euthanized when tumors reached a diameter of 20 mm or when signs of distress (including marked weight loss, lethargy, or impaired mobility) were observed. At the experimental endpoint (28 d post-injection), all remaining mice were euthanized via CO₂ inhalation followed by cervical dislocation, and tumors were excised, weighed, and processed for histology or lysate preparation. Data were analyzed using 2-tailed Student’s *t* test (GraphPad Prism v10), with significance at *P* < 0.05. This protocol, incorporating bilateral injections and Matrigel-enhanced engraftment, ensures robust tumor quantification while minimizing inter-animal variability, distinguishing it from prior unilateral or nonstromal matrix-based approaches.

### Statistical analysis

All quantitative experiments were performed in triplicate (3 independent biological replicates). Data are presented as mean ± standard deviation (SD) for technical replicates or mean ± standard error of the mean (SEM) for biological replicates, as specified. For pairwise comparisons (e.g., WT versus mutant), significance was assessed using 2-tailed Student’s *t* tests. Multi-group comparisons (e.g., dose–response assays) were analyzed by one-way analysis of variance (ANOVA) with Tukey’s post hoc correction. Correlation analyses employed Pearson’s coefficient for linear relationships or Spearman’s rank correlation for nonparametric data. Survival curves were generated via the Kaplan–Meier method, and differences were evaluated using the log-rank test.

For bioinformatics analyses, mRNA expression and clinical data were retrieved from The Cancer Genome Atlas (TCGA)–Breast Invasive Carcinoma (BRCA) and Molecular Taxonomy of Breast Cancer International Consortium (METABRIC) (via cBioPortal). Protein-level expression data were obtained from the CPTAC-BRCA project (project ID: CPTAC-BRCA; https://cptac-data-portal.georgetown.edu/), and mass spectrometry proteomics data were retrieved from the ProteomeXchange Consortium via the PRIDE repository (PXD005692, PXD008841, PXD011385). Expression levels (both mRNA and protein) were compared across clinicopathological features, including estrogen receptor (ER) status and histological grade, using the built-in tools of each platform or through customized data extraction. Kaplan–Meier survival analysis was performed using KM plotter (https://kmplot.com). RAS mutation frequencies at K117 and K147 were extracted from COSMIC (version 101), with proportions calculated relative to all missense mutations for each isoform. Statistical analyses were conducted using GraphPad Prism 10.0. A *P* value of <0.05 was considered statistically significant (*P < 0.05, **P < 0.01, ***P < 0.001). Raw data and analysis scripts are available upon request to ensure reproducibility.

## Results

### RAS is lactylated at K117 and K147 sites that are oncogenic mutations

**Fig. 1.**
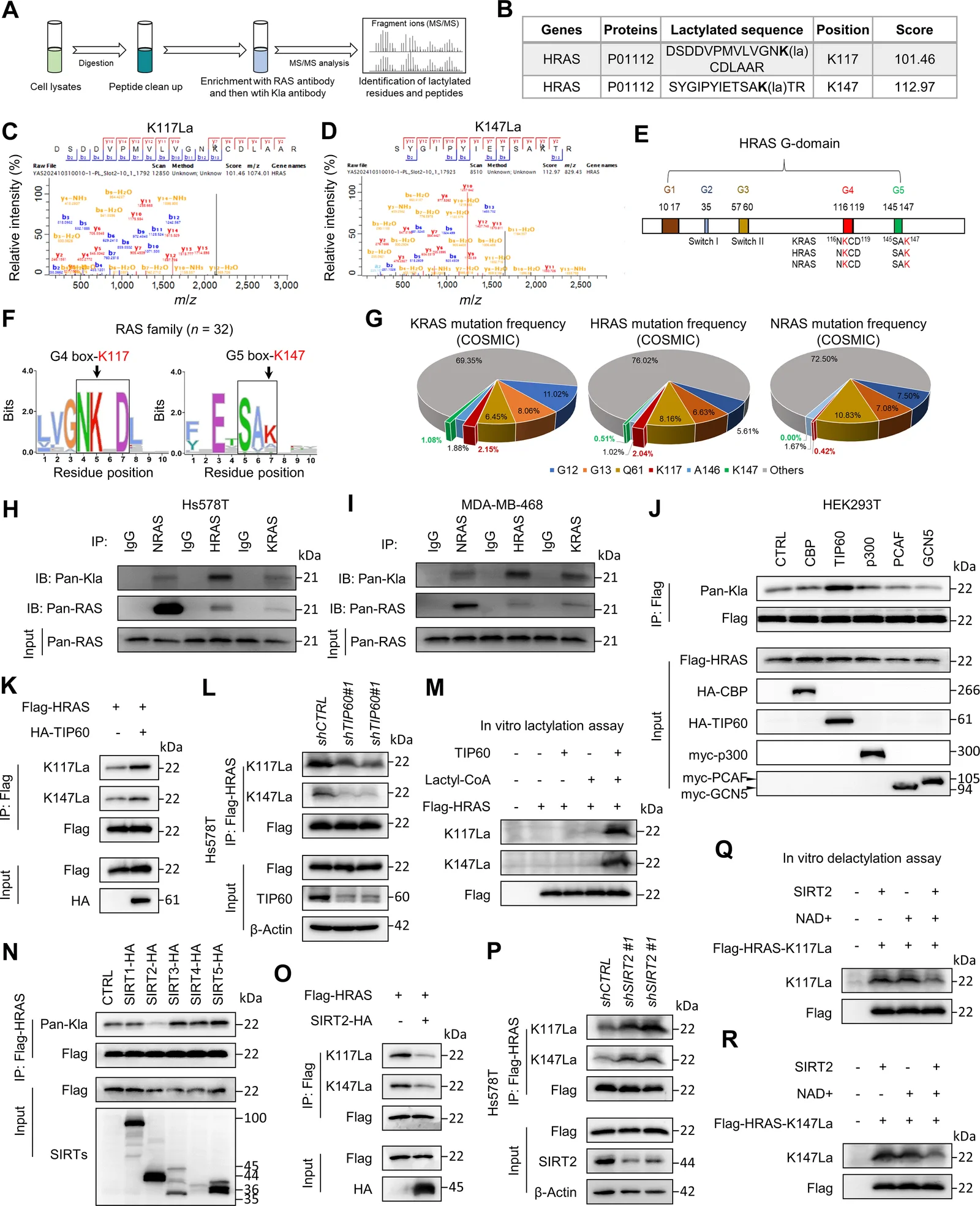
RAS undergoes lactylation at K117 and K147, 2 recurrent oncogenic mutation sites, through TIP60-mediated lactylation and SIRT2-mediated delactylation. (A) Workflow of the 3-dimensional (3D) mass spectrometry analysis used to identify HRAS lactylation sites. (B) Table showed the lactylated peptides identified by 3D mass spectrometry. (C and D) MS/MS spectra of representative HRAS peptides carrying lactylated K117 (C) and K147 (D). (E) Schematic representation of the RAS G domain. Alignment of the G4 and G5 motifs from representative RAS family proteins, with conserved lysine residues highlighted in red. (F) Sequence logo showing amino acid conservation within the G4 and G5 motifs of the RAS family. (G) Frequencies of missense mutations at the K117 and K147 hotspots in KRAS, HRAS, and NRAS in human cancers according to the COSMIC database. Red and green indicate the mutation frequencies of K117 and K147, respectively. (H and I) Hs578T (H) and MDA-MB-468 (I) cells were cultured at physiological glucose concentrations (5 mM). Endogenous RAS isoforms were immunoprecipitated from cell lysates using isoform-specific antibodies against NRAS, HRAS, or KRAS, or control IgG, as indicated. The precipitated proteins and whole-cell lysates (Input) were analyzed by Western blotting with antibodies against pan-RAS as shown. (J) HEK293T cells were cotransfected with Flag-HRAS and the indicated acyltransferases (CBP, TIP60, p300, PCAF, or GCN5) or empty vector. Flag-HRAS was immunoprecipitated and analyzed by Western blotting with a pan-Kla antibody; input lysates were probed for the acyltransferases and Flag-HRAS. (K) HEK293T cells were transfected with HRAS and/or TIP60 plasmid, and the lactylation level of HRAS was analyzed using K117La- and K147La-specific antibodies. (L) Hs578T cells expressing HRAS together with *TIP60* shRNA#1 or shRNA#2 were subjected to immunoprecipitation, and the lactylation level of HRAS was assessed using K117La- and K147La-specific antibodies. (M) In vitro lactylation assay showing the lysine lactyltransferase activity of TIP60 on HRAS-K117 and HRAS-K147. Purified Flag-HRAS was incubated with TIP60 in the presence or absence of lactyl-CoA, as indicated. Lactylation of HRAS was detected by Western blotting with K117La- and K147La-specific antibodies; Flag serves as a loading control for HRAS. (N) HEK293T cells were cotransfected with HRAS and SIRT1, SIRT2, SIRT3, SIRT4, or SIRT5 expression plasmids, and the lactylation level of HRAS was measured using the pan-Kla antibody. (O) HEK293T cells were transfected with Flag-HRAS and/or SIRT2-HA plasmid, and the lactylation level of HRAS was detected using K117La- and K147La-specific antibodies. (P) Hs578T cells expressing HRAS together with *SIRT2* shRNA#1 or shRNA#2. Following immunoprecipitation, the lactylation level of HRAS was examined using K117La- and K147La-specific antibodies. (Q and R) In vitro delactylation assay showing the delactylase activity of SIRT2 on HRAS-K117La (Q) and HRAS-K147La (R). HRAS, Harvey rat sarcoma viral oncogene homolog; NRAS, neuroblastoma RAS viral oncogene homolog; KRAS, Kirsten rat sarcoma viral oncogene homolog; K117La, lysine 117 lactylation; K147La, lysine 147 lactylation; MS, mass spectrometry; MS/MS, tandem mass spectrometry; TIP60, Tat-interactive protein 60; SIRT, sirtuin; SIRT2, sirtuin 2; shRNA, short hairpin RNA; COSMIC, Catalog of Somatic Mutations in Cancer; G4, guanine nucleotide-binding box 4; G5, guanine nucleotide-binding box 5; Kla, lysine lactylation.

Elevated RAS expression has been implicated in tumor progression [1]. Indeed, our analysis showed that KRAS and NRAS expression was tightly associated with ER^−^ breast cancer (versus ER^+^), tumor grade, and poor survival, whereas HRAS expression showed a similar trend that did not reach statistical significance (Fig. S1A to G). Lactate-derived lactylation is tightly associated with tumor progression [19]. Indeed, our analysis showed that the lactate level in breast cancer tissues was significantly higher, whereas the glucose level was significantly lower than that in normal breast tissues (Fig. S1H). In addition, the lactate level in aggressive ER^−^ breast cancer tissues was significantly higher than that in normal breast tissues and ER^+^ breast cancers with lower malignancy (Fig. S1I). Consistently, the lactate level in aggressive basal-like breast cancer cells was also significantly higher than that in the luminal subtype (Fig. S1I). To investigate whether RAS can be lactylated, we first examined HRAS (the structurally best-characterized isoform) by overexpressing Flag-HRAS in several cell lines, followed by immunoprecipitation and immunoblotting with a pan-Kla antibody. HRAS lactylation was detected under these conditions (Fig. S1J to L). In addition, we exposed HRAS-expressing HEK293T cells to various concentrations of glucose and lactate, showing that HRAS lactylation was increased by glucose and lactate in a dose-dependent manner (Fig. S1M and N). To further define the potential RAS lactylation sites, we used a specific pan-lysine lactylation (Kla) antibody and performed a mass spectrometry (MS)-based screen on lysates of HEK293T cells stably expressing HRAS (Fig. 1A). Two HRAS-derived peptides were identified, containing lactylated modifications at lysine residues (K117 and K147) in HRAS (Fig. 1B to D). Notably, K117 and K147, located in the G4 box and G5 box, respectively, were the major sites in the active region of RAS (Fig. 1E). Both K117 and K147 are highly conserved in the RAS family and even in its superfamily (Fig. 1F and Fig. S1O). Importantly, COSMIC (Catalog of Somatic Mutations in Cancer) analysis revealed K117 and K147 as recurrent missense mutation hotspots in KRAS and HRAS, whereas NRAS K147 mutations were not observed in the database (Fig. 1G). Among these sites, KRAS K117 mutations were the most frequently identified (Fig. 1G). To facilitate the specific recognition of RAS lactylation, we generated lactylation-specific antibodies against K117 and K147, respectively (designated as “K117La and K147La”). Dot blot assays were performed to characterize the specificity of the antibodies, showing that both antibodies preferentially detected lactylation at their respective sites (Fig. S2A). Mutation of K117 or K147 to arginine (or both) abolished recognition by the corresponding site-specific antibody: K117La detected only WT and K147R, whereas K147La detected only WT and K117R (Fig. S2B to D). To avoid cross-recognition caused by highly conserved or even identical sequences among RAS family members (Fig. S2E), endogenous RAS lactylation was detected in all 3 isoforms by immunoprecipitation with isoform-specific antibodies followed by lactylation antibody blotting. HRAS showed higher lactylation than KRAS and NRAS (Fig. 1H and I and Fig. S2F to H). To validate isoform-specific lactylation, we used a pan-Kla antibody to enrich lactylated proteins from cell lysates, followed by immunoblotting with HRAS-, KRAS-, and NRAS-specific antibodies. All 3 isoforms were detected, confirming endogenous lactylation of each RAS isoform (Fig. S2I). Together, these findings indicate that RAS is lactylated at K117 and K147, 2 major sites that are mutated oncogenically.

### TIP60 lactylates, whereas SIRT2 delactylates, HRAS

Aminoacyl-tRNA synthetase 1 (AARS1) is an enzyme that catalyzes the conjugation of alanine to tRNA (Ala). Recent studies have shown that AARS1 is a lactyltransferase responsible for protein lactylation [13,14]. Our study showed that *AARS1* did not affect the lactylation level of HRAS (Fig. S2J), suggesting that lactylation of different proteins may be regulated by different acyltransferases. To screen for lactyltransferases responsible for RAS lactylation, we transfected HEK293T cells with 5 known lysine acyltransferases: CREB-binding protein (CBP; KAT3A), E1A-binding protein p300 (p300; KAT3B), Tat-interactive protein 60 (TIP60; KAT5), p300/CBP-associated factor (PCAF; KAT2B), and general control non-derepressible 5 (GCN5; KAT2A), showing that ectopic expression of TIP60 increased HRAS lactylation, whereas other acyltransferases had little effect (Fig. 1J). Notably, *TIP60* expression significantly promoted, whereas knockdown of *TIP60* expression decreased, the lactylation levels of both K117 and K147 of HRAS (Fig. 1K and L). In vitro lysine lactylation confirmed that TIP60 mediated HRAS K117/K147 lactylation (Fig. 1M). These data suggest that TIP60 is a potential lysine lactyltransferase responsible for HRAS lactylation.

To identify the RAS delactylase, we coexpressed HRAS with SIRT family members (SIRT1 to SIRT5) and class I histone deacetylases (HDAC1 to HDAC3), which are established regulators of lysine deacylation [20,21], showing that HRAS lactylation was significantly reduced by SIRT2, whereas other SIRT family members and HDAC1 to HDAC3 had little effect (Fig. 1N and Fig. S2K). Furthermore, exogenous SIRT2 expression significantly decreased, whereas knockdown of SIRT2 expression promoted, the lactylation levels of both K117 and K147 of HRAS (Fig. 1O and P). In vitro delactylation assay confirmed that SIRT2 could reduce the lactylation of K117 and K147 of HRAS (Fig. 1Q and R). These data indicate that SIRT2 is a major delactylase for RAS.

### HRAS lactylation promotes its oncogenic functions

**Fig. 2.**
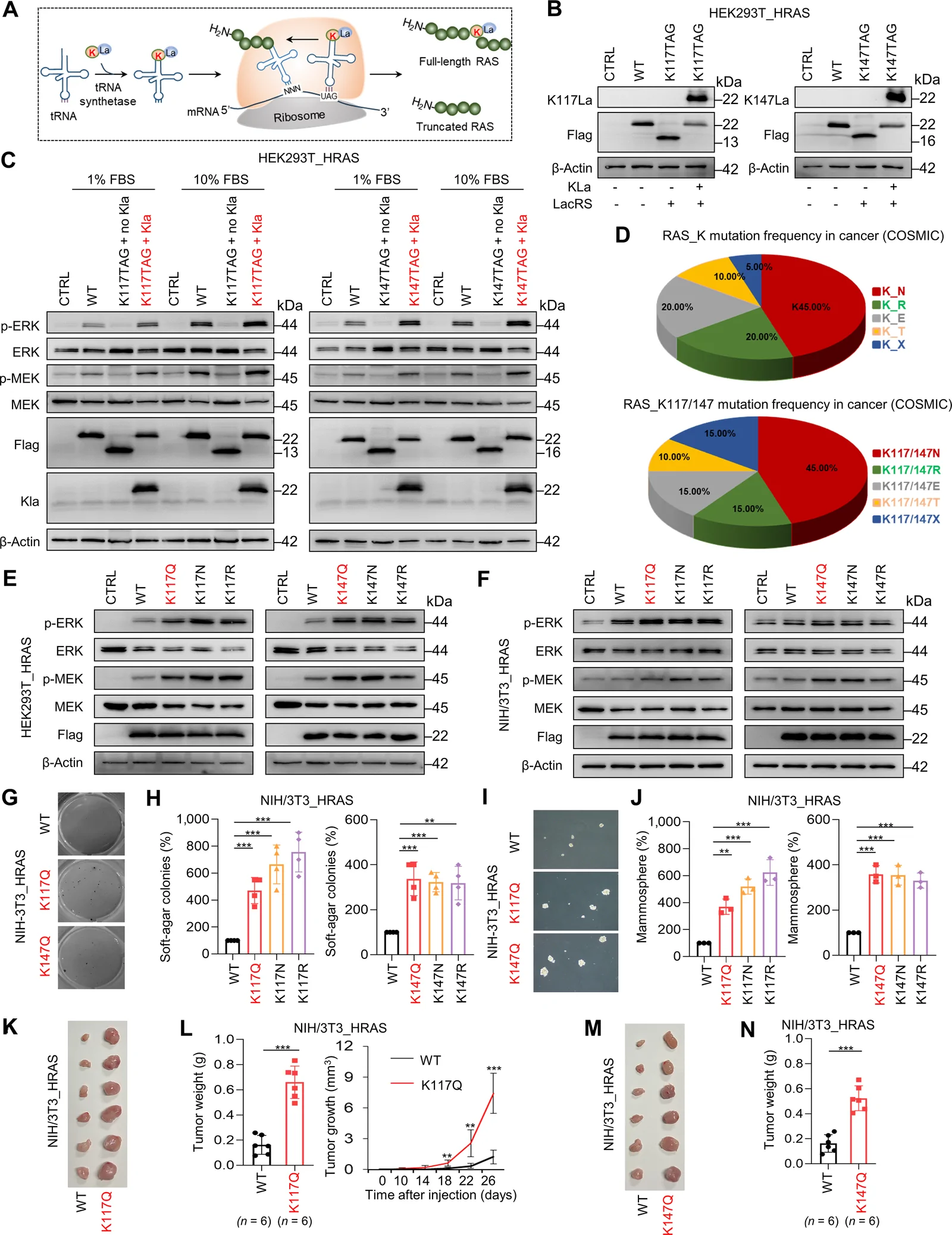
RAS lactylation promotes its activity and oncogenic functions. (A) Schematic illustration of site-specific incorporation of lactyl-lysine into recombinant HRAS using an engineered tRNA system. (B) HEK293T cells were transfected with Flag-HRAS-K117TAG or Flag-HRAS-K147TAG together with the orthogonal lactyl-tRNA synthetase (LacRS) system, and cultured with (+) or without (−) Kla. Site-specific lactylation of HRAS was assessed by Western blotting with anti-Flag and site-specific anti-K117La/K147La antibodies, as indicated. (C) Protein levels of p-ERK, ERK, p-MEK, and MEK were analyzed by Western blotting in HEK293T cells expressing WT or site-specifically lactylated HRAS cultured in medium containing 1% or 10% FBS. (D) Mutation frequencies of RAS lysine residues (top) and RAS K117/K147 (bottom) in human cancers according to the COSMIC database. (E and F) Protein levels of p-ERK, ERK, p-MEK, and MEK were analyzed by Western blotting in HEK293T (E) and NIH3T3 (F) cells expressing HRAS-WT or the indicated HRAS mutants. (G and H) Soft-agar assay was performed using NIH/3T3 cells expressing HRAS-WT or different HRAS mutants. Representative images of soft-agar colonies were shown in (G). Colony numbers were normalized to those of HRAS-WT-expressing cells, which were set to 100% (mean ± SD in 3 separate experiments; H). *P < 0.05, **P < 0.01, ***P < 0.001 by Student’s *t* test. (I and J) Mammosphere formation of NIH/3T3 cells expressing HRAS-WT or different HRAS mutants was measured. Representative images of mammospheres were shown in (I). Data were presented as a percentage of cell lines with HRAS-WT expression [mean ± SD in 3 separate experiments; (J)]. *P < 0.05, **P < 0.01, ***P < 0.001 by Student’s *t* test. (K to N) NIH/3T3 cells expressing HRAS-WT, HRAS-K117Q (K and L), or HRAS-K147Q (M and N) were injected into the mammary fat pad of nude mice. The growth of tumors was examined every 4 d. Tumor volumes were measured every 4 d, and tumor weights were determined at the study endpoint. Data were shown as mean ± SEM from 6 mice. *P < 0.05, **P < 0.01, ***P < 0.001 by Student’s *t* test. HRAS, Harvey rat sarcoma viral oncogene homolog; K117Q, lysine 117 to glutamine substitution; K147Q, lysine 147 to glutamine substitution; K117N, lysine 117 to asparagine substitution; K117R, lysine 117 to arginine substitution; K147N, lysine 147 to asparagine substitution; K147R, lysine 147 to arginine substitution; Kla, lysine lactylation; LacRS, lactyl-tRNA synthetase; WT, wild-type; p-ERK, phosphorylated extracellular signal-regulated kinase; ERK, extracellular signal-regulated kinase; p-MEK, phosphorylated mitogen-activated protein kinase kinase; MEK, mitogen-activated protein kinase kinase; COSMIC, Catalog of Somatic Mutations in Cancer; SD, standard deviation; SEM, standard error of the mean.

To unambiguously determine the effect of lactylation on RAS, we employed a site-specific lactylation system of genetic code expansion to incorporate lactyl-lysine into recombinant RAS to generate site-specific fully lactylated proteins in vitro (Fig. 2A). We tested the feasibility of this system. Specifically, HEK293T cells were cotransfected with genes encoding Flag-RAS (HRAS or KRAS) or C-terminally enhanced GFP (EGFP)-tagged RAS containing an amber mutation at K117 or K147, together with the PylRS/PylT pair, and cultured with or without lactyl-lysine. Immunoblot analysis revealed specific bands only in the presence of lactyl-lysine (Fig. 2B and Fig. S3A), confirming site-specific incorporation of the modification into RAS.

To analyze the effect of HRAS lactylation on downstream signaling pathways, we examined the phosphorylation of ERK and MEK in starved and nonstarved HEK293T clones. Both HRAS K117 and K147 lactylation generated by the site-specific lactylation system led to increased phosphorylation of both ERK and MEK compared with HRAS-WT under starvation or nonstarvation conditions (Fig. 2C), whereas in the absence of lactyl-lysine, the truncated RAS 1 to 116 and 1 to 146 amino acid fragments produced by the system that lack the CAAX motif critical for HRAS activity exhibited no detectable activation of ERK signaling (Fig. 2C). In order to reduce the experimental burden associated with the site-specific lactylation system, subsequent experiments no longer included the condition without lactyl-lysine. In addition, we analyzed the mutation frequencies at K117 and K147 in cancers using the COSMIC database. COSMIC analysis revealed that K117 and K147 shared a similar mutation spectrum, with asparagine (N), arginine (R), and glutamic acid (E) being the 3 most frequent substitutions at both residues, among which N was the most prevalent (Fig. 2D). In subsequent experiments, we selected the most frequent cancer-associated mutations (K117N, K117R, K147N, and K147R) for comparative analysis with lactylation. We generated HEK293T and NIH/3T3 clones expressing HRAS with cancer-associated mutations (K117N, K117R, K147N, or K147R) or the lactylation-mimetic mutants (K117Q or K147Q). Consistent with site-specifically lactylated HRAS, these mutants showed increased ERK and MEK phosphorylation compared with the HRAS-WT (Fig. 2E and F). To compare genuine lactylation with canonical oncogenic mutations, we used our site-specific lactylation system to express HRAS-K117La or HRAS-K147La alongside HRAS-WT and HRAS-G12D. GST-RBD pull-down and coimmunoprecipitation assays showed that K117La, K147La, and G12D all significantly increased GTP-bound RAS compared to WT (Fig. S3B and C) and similarly elevated p-ERK/p-MEK levels (Fig. S3D). Similar results were also observed for KRAS (Fig. S3E and F). In addition, KRAS lactylation at K117/K147 was enhanced by TIP60 overexpression and SIRT2 knockdown, but reduced by TIP60 knockdown and SIRT2 overexpression (Fig. S3G and H), indicating that the TIP60–SIRT2 regulatory axis is conserved across RAS isoforms. HRAS is one of the most studied RAS isoforms in terms of mechanistic and functional studies, largely due to its well-characterized structure and its role in various cellular processes, making it a preferred subject for many mechanistic studies [2,8,22]. Based on the functional similarities between HRAS and KRAS, the higher lactylation level of HRAS, and the advantages of structural analysis, we chose HRAS for subsequent experiments.

Mutagenesis can be elegantly used to mimic PTMs, such as replacing K with Q to mimic lysine acetylation [23]. However, there is currently no recognized amino acid that mimics lactylation. The side-chain ends of lysine acetylation, lysine lactylation, and glutamine are composed of a carbonyl group (-C=O) and a nonpolar methyl group (-CH3), a carbonyl group (-C=O) and a polar hydroxyethyl group (-CH3CHOH), and a carbonyl group (-C=O) and a polar amino group (-NH2), respectively. Based on the polar characteristics of the side-chain groups of both lysine lactylation (but not acetylation) and glutamine, replacing K with Q may be a better choice to mimic lysine lactylation than acetylation. Indeed, our data show that the functional trends of lysine lactylation simulated by Q replacing K are consistent with those of the lactylated proteins generated by the site-specific lactylation system (Fig. 2C to F and Fig. S3E and F). For subsequent experiments with a longer duration, we mimicked lysine lactylation using Q to substitute K. To evaluate the potential malignant transformation function of HRAS lactylation, HRAS lactylation-mimicking variants (K117Q or K147Q) and tumor-associated mutant variants (K117N, K117R, K147N, or K147R) were ectopically expressed in NIH/3T3 cells, showing that these cells formed more colonies or mammospheres when grown in soft agar, or under mammosphere-forming conditions than those with HRAS-WT (Fig. 2G to J). To further confirm that K117 and K147 lactylation are oncogenic PTMs, NIH/3T3 cells expressing mutants of HRAS K117Q or K147Q were injected subcutaneously into nude mice. As expected, cells expressing HRAS K117Q or K147Q exhibited significantly increased tumor growth compared with cells expressing HRAS-WT (Fig. 2K to N and Fig. S3I). Together, these findings indicate that RAS lactylation enhances its oncogenic functions.

### HRAS lactylation promotes its activity by disrupting its GTP interaction and impairing intrinsic GTPase activity

**Fig. 3.**
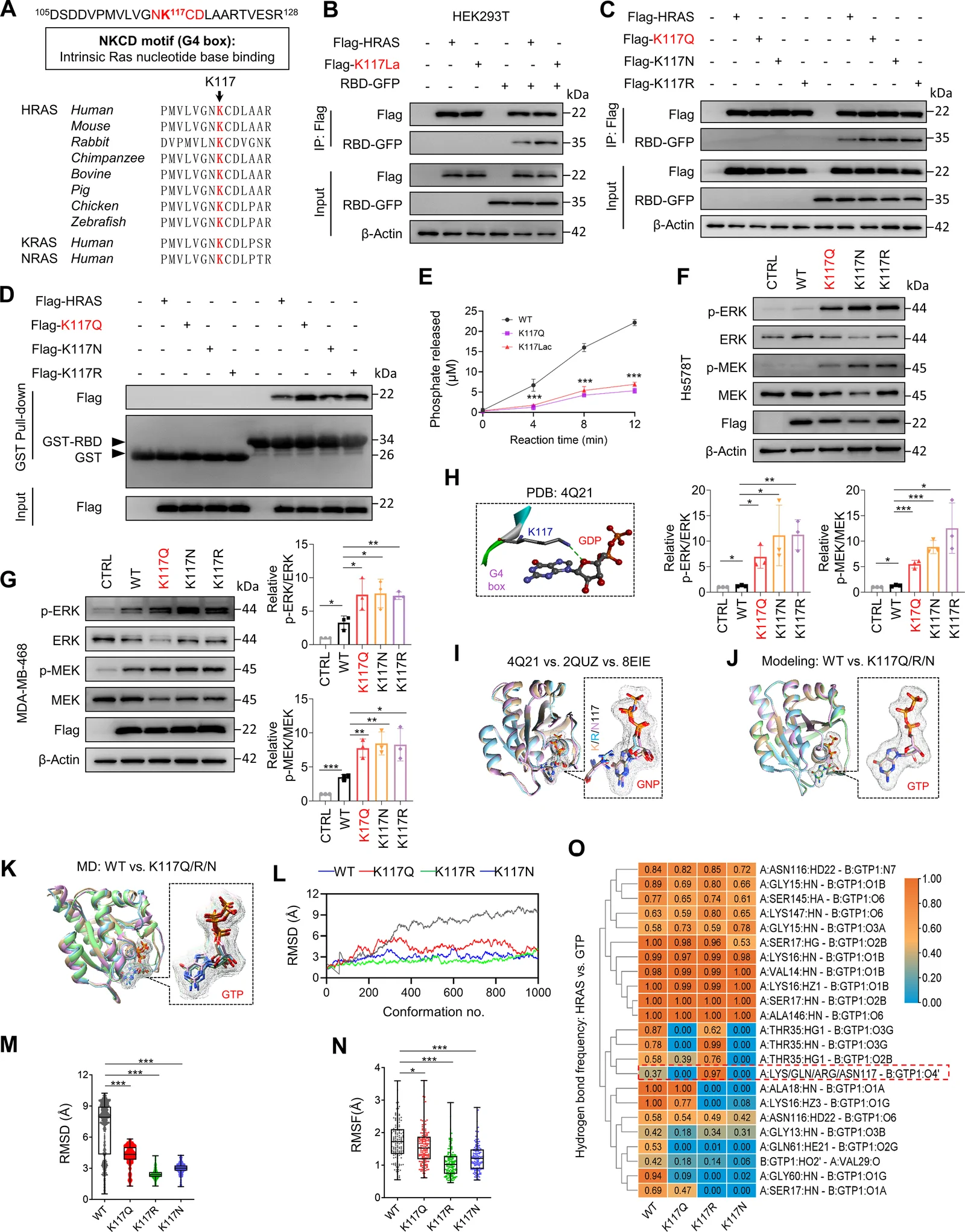
K117 lactylation contributes to accumulation of the active GTP-bound RAS. (A) Multiple sequence alignment showing conservation of HRAS K117 and its flanking residues across different species. (B and C) RBD-GFP was cotransfected with WT or lactylated HRAS (B) or HRAS mutants (C) in HEK293T cells, and immunoprecipitated RBD-GFP was analyzed by Western blotting. (D) HRAS-WT or different mutants were expressed in HEK293T cells, and RAS activity was determined using a GST-RBD pull-down assay. (E) WT-, lactylated, and mutant HRAS proteins were loaded with GTP, and intrinsic GTP hydrolysis was measured by continuously monitoring phosphate release using a colorimetric assay. Released phosphate concentration was plotted over time. (F and G) Protein levels of p-ERK, ERK, p-MEK, and MEK in Hs578T (F) or MDA-MB-468 cells (G) expressing HRAS-WT or different mutants was examined by Western blotting. Quantification of the immunoblots is shown as bar graphs (mean ± SD). (H) Structural view of interactions between K117 in the G4 motif and GDP in HRAS (PDB: 4Q21). Hydrogen bonds were indicated by dashed lines. (I) Superposition of crystal structures of HRAS-WT (PDB: 4Q21, tan), the K117R mutant (PDB: 2QUZ, sky blue), and the K117N mutant (PDB: 8EIE, pink). Enlarged views of the nucleotide-binding site were shown on the right. (J and K) Overlay of HRAS-WT (tan), K117Q (sky blue), K117R (pink), and K117N (light green) mutants: superposition of modeled structures (J) or representative snapshots from MD simulations (K). A magnified overlay of GTP structure was shown on the right. (L and M) Overlay of GTP RMSD trajectories (L) and corresponding histogram of RMSD values (M) for HRAS-WT, K117Q, K117R, and K117N mutants from molecular dynamics (MD) simulations. *P < 0.05, **P < 0.01, ***P < 0.001 by Student’s *t* test. (N) Histogram of protein backbone Cα RMSF values obtained from MD simulations for HRAS-WT, K117Q, K117R, and K117N mutants. *P < 0.05, **P < 0.01, ***P < 0.001 by Student’s *t* test. (O) Heatmap showing hydrogen bond occupancies between GTP and HRAS-WT or the indicated mutants. Occupancy was calculated as the fraction of simulation time during which each hydrogen bond was present. HRAS, Harvey rat sarcoma viral oncogene homolog; RBD, RAS-binding domain; GFP, green fluorescent protein; WT, wild-type; GST, glutathione *S*-transferase; GTP, guanosine triphosphate; GDP, guanosine diphosphate; p-ERK, phosphorylated extracellular signal-regulated kinase; ERK, extracellular signal-regulated kinase; p-MEK, phosphorylated mitogen-activated protein kinase kinase; MEK, mitogen-activated protein kinase kinase; G4, guanine nucleotide-binding box 4; PDB, Protein Data Bank; K117Q, lysine 117 to glutamine substitution; K117R, lysine 117 to arginine substitution; K117N, lysine 117 to asparagine substitution; MD, molecular dynamics; RMSD, root mean square deviation; RMSF, root mean square fluctuation; Cα, alpha carbon.

RAS K117 is highly evolutionarily conserved from zebrafish to humans (Fig. 3A). Based on the fact that K117 is critical for the binding of RAS to GDP or GTP, we fused the RAS-binding domain (RBD) of human Raf1 (amino acids 1 to 149) that preferentially binds to GTP-loaded RAS, to GFP and GST, respectively, and then evaluated the effects of lactylation and mutation on binding ability by coimmunoprecipitation and pull-down assays. As expected, K117-lactylated HRAS produced by the site-specific lactylation system significantly enhanced the binding ability of HRAS to RBD (Fig. 3B). Consistently, all the K117 mutants also resulted in increased binding of HRAS to the RBD (Fig. 3C and D). RAS has intrinsic GTPase activity, the inactivation of which activates downstream signaling [1]. To determine the effects of K117 lactylation and oncogenic mutations on intrinsic GTP hydrolysis, we measured phosphate release. The intrinsic hydrolysis ability of K117 lactylation was comparable to that of the K117Q mutant, but significantly lower than that of HRAS-WT (Fig. 3E). GTP pull-down and nucleotide exchange analysis showed that neither lactylation nor the K117Q mutation altered nucleotide-binding affinity or exchange rates of HRAS (Fig. S4A and B). We further examined the effects of HRAS K117 mutants on downstream signaling pathways in breast cancer cell lines, including Hs578T and MDA-MB-468 cells. Notably, these mutants significantly increased ERK and MEK phosphorylation compared with HRAS-WT (Fig. 3F and G).

Structural analysis showed that the side-chain amino group of K117 in RAS formed a hydrogen bond with the ribose of guanine nucleotide (Fig. 3H). The compilation of RAS crystal structures indicated that WT- and K117-mutated RAS (RAS K117R and RAS K117N) had extremely similar conformations in GTP and its binding region (Fig. 3I and J and Fig. S4C and D), whereas random snapshots in the MD trajectory showed clear changes in the RAS-GTP binding conformation (Fig. 3K and Fig. S4E to G), indicating that it is difficult to analyze the potential mechanistic and functional differences between WT and mutant RAS by these static structures, and MD simulations make up for this deficiency to a certain extent. To gain insight into the potential mechanisms by which K117 lactylation promoted RAS activity, MD simulations were performed to determine how K117 lactylation affected the structure of HRAS. RMSD analysis was performed to evaluate the structural stability of the protein–ligand complex. RMSD analysis showed that HRAS K117 mutants (K117Q/R/N) had significantly lower RMSD values than HRAS-WT, suggesting a more stable protein–GTP complex (Fig. 3L and M and Fig. S4H to J). RMSF analysis provides the flexibility and fluctuation of each residue in the protein structure. Notably, the RMSF values ​​of all K117 mutants were lower than those of HRAS-WT (Fig. 3N and Fig. S4K to N).

Hydrogen bonds play a crucial role in the specificity and directionality of molecular recognition between RAS and its ligand GTP. In RAS, GTP forms hydrogen bonds with G13, G15, K16, S17, and A18 in the P-loop (G1) of HRAS, T35 in the G2 region, G60 and Q61 in the G3 region, N116 and K117 in the G4 box, and S145, A146, and K147 in the G5 box, respectively [24]. K117 is positively charged before lactylation, and its lactylation neutralizes the charge of the residue, destroying its favorable interaction with GTP. Therefore, the effect of K117 lactylation on RAS may be due to the perturbation of electrostatic interactions. Indeed, our data showed that compared with HRAS-WT, the lactylation-mimicking mutation K117Q and the tumor-associated mutation K117N lost the hydrogen bond with GTP, whereas the positively charged K117R increased its frequency of hydrogen bonding with GTP (Fig. 3O and Fig. S4O). In addition to the effect of hydrogen bonding between residue 117 and GTP, these K117 mutations abolished or significantly reduced the occupancy of hydrogen bonds between GTP and residues such as G13, K16, S17, T35, and G60, which are closely related to intrinsic RAS-GTPase activity (Fig. 3O and Fig. S4O). These data suggest that lactylation or mutation of HRAS-K117 leads to impairment or loss of its GTPase activity.

**Fig. 4.**
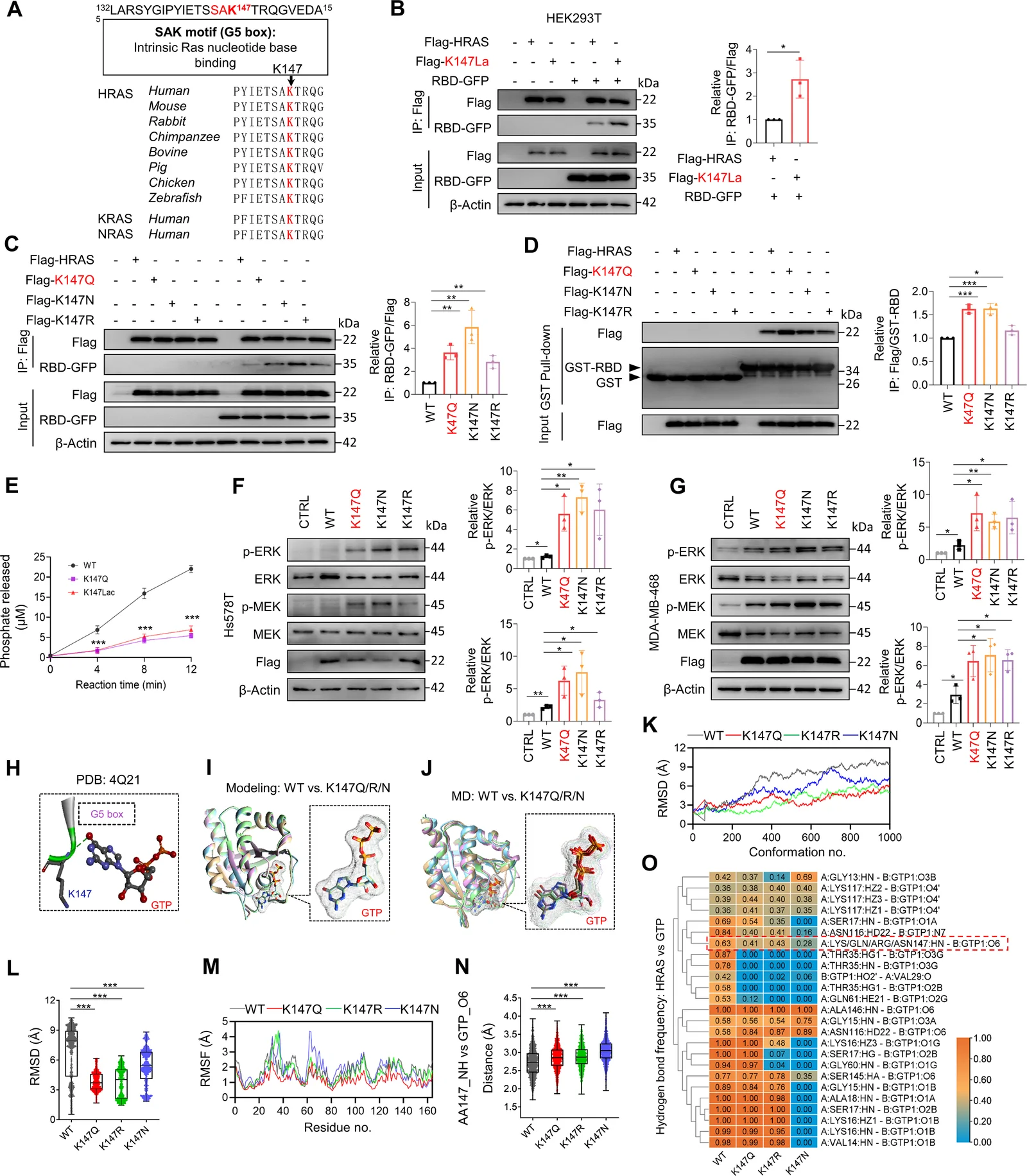
K147 lactylation contributes to accumulation of the active GTP-bound RAS. (A) Alignment of HRAS K147 and adjacent protein sequences in different organisms. (B and C) HRAS-WT, lactylated HRAS (B), or different HRAS mutants (C) were expressed in HEK293T cells, and immunoprecipitated RBD-GFP was analyzed by Western blotting. Quantification of the immunoblots is shown as bar graphs (mean ± SD). (D) HRAS-WT or different mutants were expressed in HEK293T cells, and HRAS activity was assessed using a GST-RBD pull-down assay. Quantification of the immunoblots is shown as bar graphs (mean ± SD). (E) WT-, lactylated, and mutant HRAS proteins were loaded with GTP, and intrinsic GTP hydrolysis was measured by continuous colorimetric detection of released phosphate. Phosphate concentration was plotted as a function of time. (F and G) Protein levels of p-ERK, ERK, p-MEK, and MEK were analyzed by Western blotting in Hs578T (F) and MDA-MB-468 (G) cells expressing HRAS-WT or the indicated mutants. Quantification of the immunoblots is shown as bar graphs (mean ± SD). (H) Structural interactions involving K147 within the G5 motif of GDP-bound HRAS (PDB: 4Q21). Hydrogen bonds are shown as dashed lines. Hydrogen bond interactions were shown as dashed lines. (I and J) Structural overlays of HRAS-WT (tan), K147Q (sky blue), K147R (pink), and K147N (light green) after structural modeling (I) or from representative snapshots of the MD trajectories (J). Enlarged views of the GTP-binding site were shown on the right. (K and L) Overlay (K) and histogram (L) of GTP RMSD values from MD simulations of HRAS-WT and the indicated mutants. *P < 0.05, **P < 0.01, ***P < 0.001 by Student’s *t* test. (M) Overlay of protein backbone Cα RMSF profiles from MD simulations of HRAS-WT and the indicated mutants. (N) Distance between the backbone NH group of residue 147 and the O6 atom of GTP during MD simulations of HRAS-WT and the indicated mutants. *P < 0.05, **P < 0.01, ***P < 0.001 by Student’s *t* test. (O) Heatmap showing hydrogen bond interactions between GTP and HRAS-WT or the indicated mutants. Occupancy was calculated as the fraction of simulation time during which each hydrogen bond was present. HRAS, Harvey rat sarcoma viral oncogene homolog; RBD, RAS-binding domain; GFP, green fluorescent protein; WT, wild-type; GST, glutathione *S*-transferase; GTP, guanosine triphosphate; GDP, guanosine diphosphate; p-ERK, phosphorylated extracellular signal-regulated kinase; ERK, extracellular signal-regulated kinase; p-MEK, phosphorylated mitogen-activated protein kinase kinase; MEK, mitogen-activated protein kinase kinase; G5, guanine nucleotide-binding box 5; PDB, Protein Data Bank; K147Q, lysine 147 to glutamine substitution; K147R, lysine 147 to arginine substitution; K147N, lysine 147 to asparagine substitution; MD, molecular dynamics; RMSD, root mean square deviation; RMSF, root mean square fluctuation; Cα, alpha carbon; NH, amide group; O6, oxygen 6 of guanine.

RAS K147 is also highly evolutionarily conserved (Fig. 4A). Due to the important role of K147 on RAS and GDP or GTP, we evaluated the impact of K147 lactylation and mutations on binding ability through coimmunoprecipitation and pull-down analysis, showing that both K147 lactylation and K147 mutations significantly enhanced the binding ability of HRAS and RBD (Fig. 4B to D). Phosphate assay kit analysis showed that the intrinsic GTP hydrolyzing capacity of K147 lactylation or K147Q mutant was significantly lower than that of HRAS-WT (Fig. 4E). Nucleotide exchange analysis showed that neither lactylation nor the K147Q mutation altered nucleotide exchange rates of HRAS (Fig. S5A). In addition, these K147 mutants also significantly increased ERK and MEK phosphorylation compared with HRAS-WT (Fig. 4F and G).

Structural analysis showed that the NH group of the backbone from K147 forms a hydrogen bond with the guanine ring (Fig. 4H). RAS structural modeling showed that the HRAS-WT and HRAS K147 mutants (K147Q/R/N) had very similar conformations in GTP and its binding region, whereas random snapshots in the MD trajectory showed clear changes in the RAS-GTP binding conformation (Fig. 4I and J and Fig. S5B to D). RMSD analysis revealed that all HRAS K147 mutants (K147Q/R/N) showed significantly less structural deviation than RAS-WT (Fig. 4K and L and Fig. S5E to G). The RMSF value of the K147 mutation was unchanged or slightly lower than that of HRAS-WT (Fig. 4M and Fig. S5H to K). Since K147 forms hydrogen bonds with the guanine ring through the NH group on the backbone rather than the amino group on the side chain, lactylation of the K147 side chain may not affect the formation of hydrogen bonds. To further understand whether K147 lactylation affected the hydrogen bond formation of amino acid residue 147 to GTP, we analyzed the distance between the NH group of backbone from amino acid residue 147 and O6 of GTP during MD. Our data showed that the distance in all these HRAS K147 mutants were significantly increased relative to that in HRAS-WT (Fig. 4N and Fig. S5L and M). In addition, beyond the hydrogen bonding between amino acid residue 147 and GTP, these K147 mutants also eliminated or significantly reduced the hydrogen bonding occupancy between GTP and residues S17, T35, N61, and Q61 (Fig. 4O and Fig. S5N), which is tightly related to the intrinsic GTPase activity of RAS. These data suggest that K147 lactylation or mutation compromises the intrinsic GTPase activity of RAS, ultimately leading to RAS activation.

### K147 lactylation stabilizes HRAS by competitively antagonizing protein ubiquitination

**Fig. 5.**
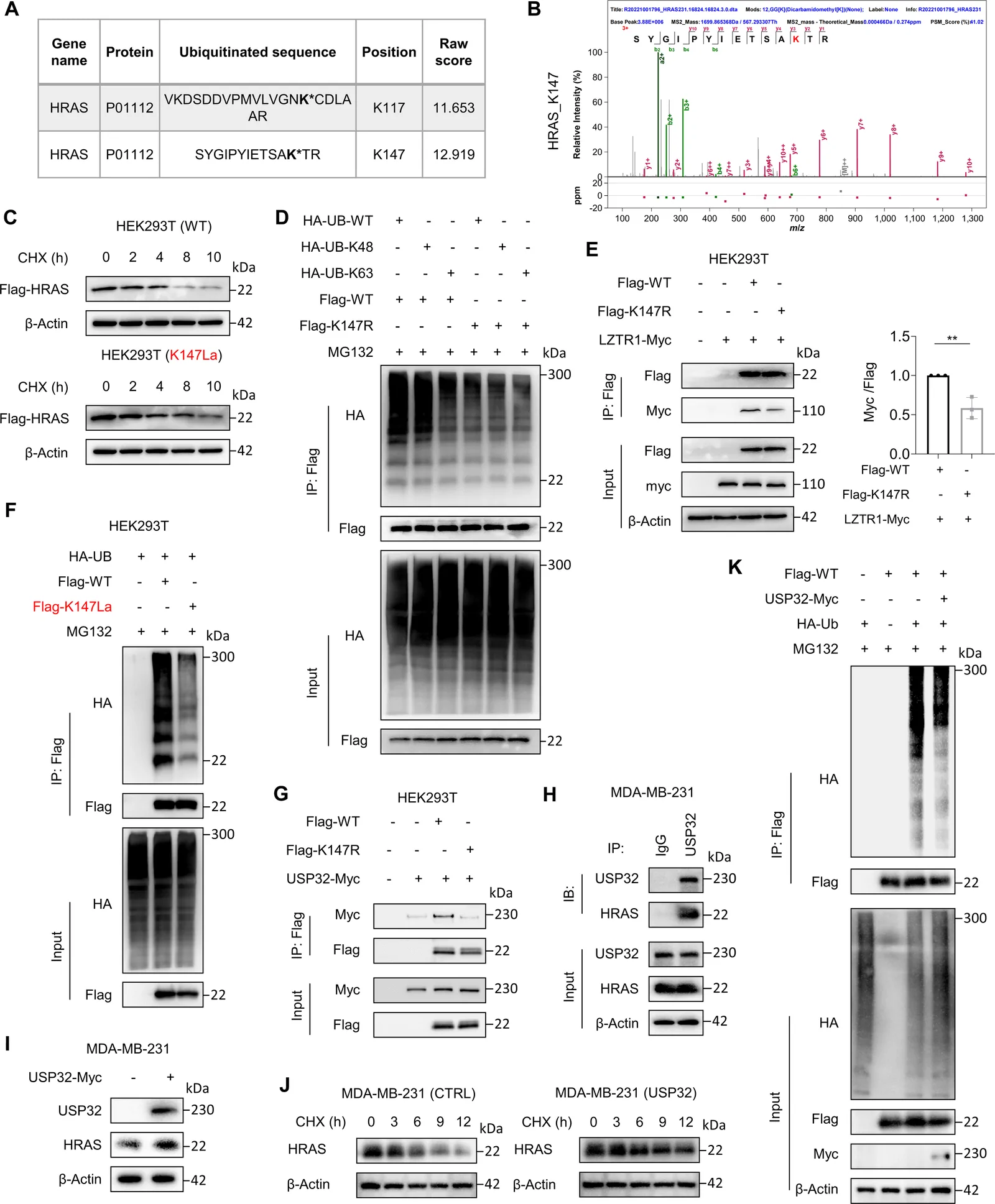
K147 lactylation stabilizes HRAS by competitively antagonizing protein ubiquitination. (A) Summary of ubiquitinated HRAS peptides identified by mass spectrometry. (B) Representative MS/MS spectrum of the HRAS peptide containing ubiquitinated K147. (C) HEK293T cells expressing WT (top) or site-specifically lactylated HRAS (bottom) were treated with 50 μg/ml CHX for the indicated times after 48 h of expression, and HRAS protein levels were analyzed by immunoblotting. (D) HEK293T cells were cotransfected with the indicated plasmids, and K48- and K63-linked ubiquitination of HRAS was assessed. (E) HEK293T cells expressing HRAS-WT and/or the K147R mutant together with LZTR1 were subjected to Flag immunoprecipitation, followed by immunoblot analysis of protein interactions. Quantification of the immunoblots is shown as bar graphs (mean ± SD). (F) HEK293T cells were cotransfected with the indicated plasmids, and ubiquitination of WT and site-specifically lactylated HRAS was analyzed. (G) HEK293T cells expressing HRAS-WT and/or the K147R mutant together with USP32 were subjected to Flag immunoprecipitation, followed by immunoblot analysis of protein interactions. (H) MDA-MB-231 cell lysates were immunoprecipitated with an anti-USP32 antibody, and coimmunoprecipitated endogenous HRAS was detected by immunoblotting. (I) HRAS and USP32 protein levels were analyzed by immunoblotting in MDA-MB-231 cells with stable empty vector or USP32 expression. (J) MDA-MB-231 cells stably expressing the empty vector or USP32 were treated with 25 μg/ml CHX for the indicated times, and HRAS protein levels were analyzed by immunoblotting. (K) HEK293T cells were cotransfected with the indicated plasmids, and HRAS ubiquitination was analyzed by ubiquitination assay. HRAS, Harvey rat sarcoma viral oncogene homolog; MS, mass spectrometry; MS/MS, tandem mass spectrometry; CHX, cycloheximide; WT, wild-type; K48, lysine 48; K63, lysine 63; LZTR1, leucine-zipper-like transcriptional regulator 1; USP32, ubiquitin-specific protease 32; K147R, lysine-to-arginine substitution at residue 147; IP, immunoprecipitation; Ub, ubiquitin.

It has been reported that the stability of RAS proteins is associated with protein degradation via ubiquitination [25]. To identify the potential RAS ubiquitination sites, we performed an MS-based screen on lysates of HEK293T cells stably expressing HRAS. Several HRAS-derived peptides were identified, containing ubiquitination at K117 and K147 in HRAS (Fig. 5A and B and Fig. S6A). Cycloheximide (CHX) assay and ubiquitination assay showed that HRAS was an unstable protein; however, RAS lactylation and K147R, but not K117R, significantly enhanced its protein stability (Fig. 5C and Fig. S6B). K48-linked ubiquitin chains target proteins for proteasomal degradation, whereas non-K48-linked ubiquitin chains, such as K63-linked polyubiquitin chains, mainly regulate protein activity and other functions [26,27]. We found that HRAS was predominantly modified by K48-linked, rather than K63-linked, polyubiquitin chains (Fig. 5D), indicating that K147 ubiquitination is mainly involved in proteasome-mediated protein degradation. Leucine-zipper-like transcriptional regulator 1 (LZTR1) is a substrate-specific adapter of a BCR (BTB-CUL3-RBX1) E3 ubiquitin–protein ligase complex that mediates RAS ubiquitination [25]. Ubiquitination assays with LZTR1 overexpression showed that both K147R and K147La had much less ubiquitination than WT, indicating that lactylation blocks ubiquitin conjugation at K147 (Fig. S6C). Immunoprecipitation experiments further confirmed that HRAS-WT could bind to LZTR1, whereas both K147La and K147R significantly reduced this binding (Fig. 5E and Fig. S6D). To clarify the relationship between K147 lactylation and HRAS protein stability, we performed ubiquitination analysis. The data showed that compared with HRAS-WT, K147 lactylation significantly reduced HRAS ubiquitination level (Fig. 5F), suggesting that HRAS K147 lactylation competitively blocks ubiquitination at this site. Ubiquitin-specific protease 32 (USP32) is a deubiquitinase that can remove conjugated ubiquitin from target proteins [28]. Analysis of the previous protein–protein interaction database [29] showed that USP32 was a putative RAS-interacting deubiquitinase (Fig. S6E). Clinical data analysis showed that abnormally high expression of *USP32* was closely correlated with breast cancer, poor survival, and high *HRAS* expression (Fig. S6F to H). To determine whether it was a potential RAS-interacting deubiquitinase, we determined the interaction between USP32 and HRAS by immunoprecipitation, confirming the interaction between USP32 and HRAS (Fig. 5G and H). We further tested the effect of USP32 on HRAS, showing that *USP32* expression remarkably increased HRAS protein levels (Fig. 5I and Fig. S6I). Consistently, CHX assay and ubiquitination assay showed that *USP32* expression significantly improved HRAS protein stability (Fig. 5J and K and Fig. S6J). These findings indicate that USP32 is a major RAS deubiquitinase.

### RAS lactylation is tumor-associated, and inhibition of RAS lactylation sensitizes cancer cells to drugs targeting RAS mutations

**Fig. 6.**
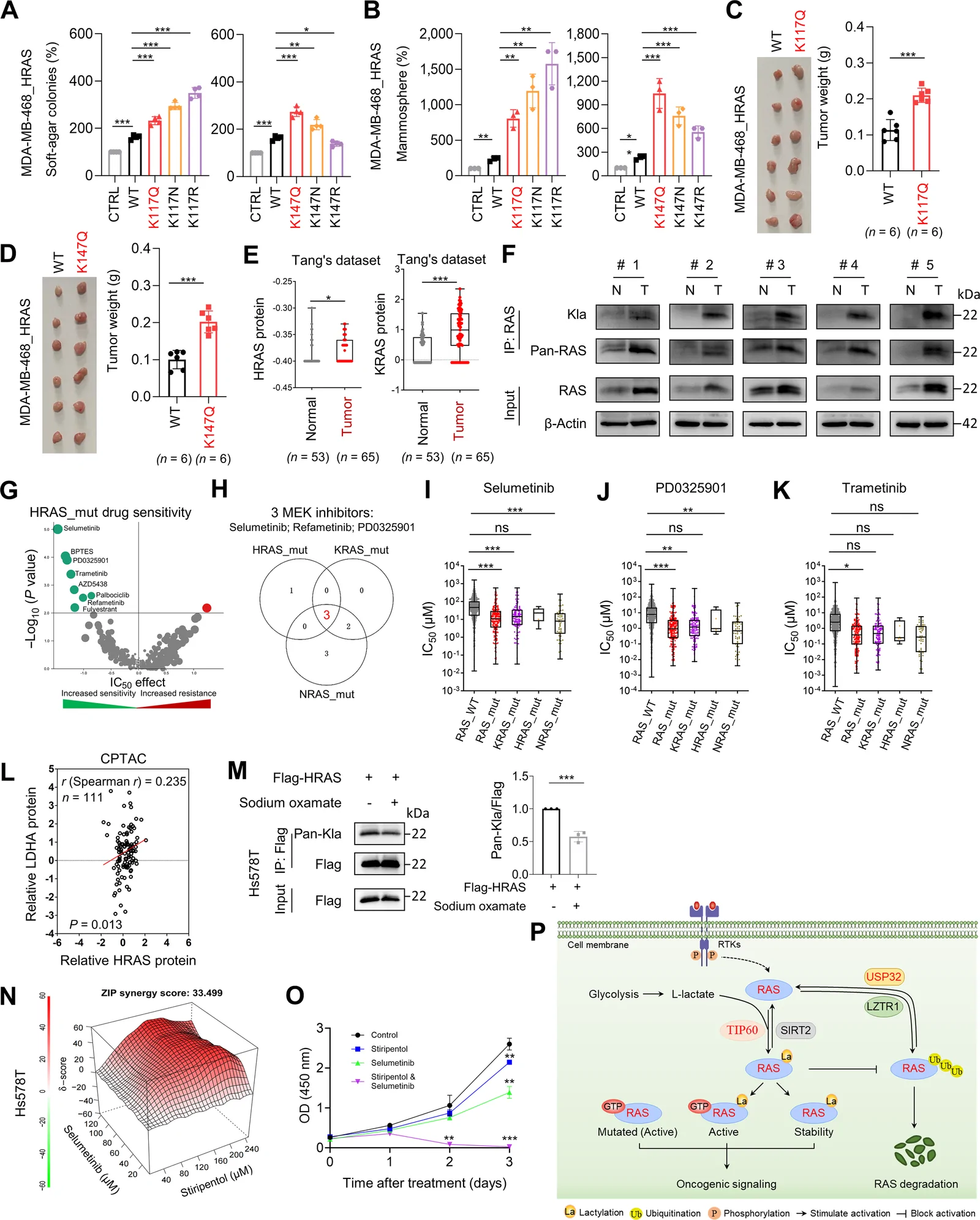
RAS lactylation was tumor-associated, and inhibition of RAS lactylation sensitizes cancer cells to drugs targeting RAS mutations. (A) Soft-agar assay was performed using MDA-MB-468 cells expressing HRAS-WT or different HRAS mutants. Colony formation was normalized to the empty vector control (mean ± SD in 3 separate experiments). *P < 0.05, **P < 0.01, ***P < 0.001 by Student’s *t* test. (B) Mammosphere formation by MDA-MB-468 cells expressing HRAS-WT or different HRAS mutants was measured. Data were presented as percentages relative to the empty vector control (mean ± SD in 3 separate experiments). *P < 0.05, **P < 0.01, ***P < 0.001 by Student’s *t* test. (C and D) MDA-MB-468 cells expressing HRAS-WT, HRAS-K117Q (C), or HRAS-K147Q (D) were injected into the mammary fat pad of nude mice. Tumor growth was monitored every 4 d. Tumor volume and final tumor weight were measured. Data are presented as mean ± SEM (*n* = 6 mice per group). *P < 0.05, **P < 0.01, ***P < 0.001 by Student’s *t* test. (E) RAS protein expression in paired normal and tumor tissues from Tang’s dataset. (F) Paired human breast tumor (T) and adjacent normal (N) tissues from breast cancer patients were analyzed. Tissue lysates were subjected to Western blotting for pan-RAS. For pan-RAS lactylation detection, lysates were immunoprecipitated with a pan-RAS antibody and then immunoblotted with a RAS K117/K147 lactylation-specific antibody. (Analysis of additional samples is shown in Fig. S8A.) (G) Volcano plot showing differential drug sensitivity associated with HRAS mutations (increased sensitivity in green and decreased sensitivity in red). (H) Venn diagram showing drugs exhibiting increased sensitivity across HRAS, KRAS, and NRAS mutations. (I to K) Drug sensitivity of cell lines harboring WT or mutant HRAS, KRAS, or NRAS to selumetinib (I), PD0325901 (J), and refametinib (K) was analyzed using the GDSC database. Cell lines were grouped as RAS-WT (no RAS mutations) or RAS-mutant (any missense mutation in HRAS, KRAS, or NRAS). (L) Correlation analysis between HRAS and LDHA protein expression in the CPTAC dataset. The relative level of HRAS was plotted against that of LDHA. (M) Hs578T cells expressing HRAS-WT were treated with or without sodium oxamate (20 mM) for 24 h, followed by immunoprecipitation. The Kla levels of precipitated protein were analyzed by Western blotting. Quantification of the immunoblots is shown as bar graphs (mean ± SD). (N) Hs578T cells were treated with increasing concentrations of stiripentol (LDHA inhibitor) and selumetinib (MEK inhibitor) alone or in combination for 48 h. Cell viability was assessed, and synergy was quantified using the ZIP model; the synergy score was displayed in the synergy matrix. (O) Cell viability of the indicated cells was analyzed following treatment with selumetinib (0.08 mM), stiripentol (0.12 mM) or the combination for the indicated times. Data were presented as mean ± SD in 3 separate experiments. *P < 0.05, **P < 0.01, ***P < 0.001 by Student’s *t* test. (P) A proposed model to illustrate mechanism of lactylation-mediated RAS activation and protein stability, promoting malignant transformation and tumorigenesis (please see Discussion). HRAS, Harvey rat sarcoma viral oncogene homolog; KRAS, Kirsten rat sarcoma viral oncogene homolog; NRAS, neuroblastoma RAS viral oncogene homolog; WT, wild-type; SD, standard deviation; SEM, standard error of the mean; K117Q, lysine 117 to glutamine substitution; K147Q, lysine 147 to glutamine substitution; K117N, lysine 117 to asparagine substitution; K117R, lysine 117 to arginine substitution; K147N, lysine 147 to asparagine substitution; K147R, lysine 147 to arginine substitution; GDSC, Genomics of Drug Sensitivity in Cancer; TCGA, The Cancer Genome Atlas; LDHA, lactate dehydrogenase A; Kla, lysine lactylation; IP, immunoprecipitation.

To investigate the biological consequences of HRAS lactylation and tumor-associated mutations, HRAS lactylation-mimicking variants and tumor-associated mutant variants were ectopically expressed in multiple tumor cell lines. Compared with HRAS-WT, most HRAS mutants enhanced colony and mammosphere formation, with cell-context-dependent variations. In Hs578T cells, all mutants promoted both phenotypes (Fig. S7A and B). In MDA-MB-468 cells, K147R reduced colony formation but still enhanced mammosphere formation, whereas all other mutants promoted both (Fig. 6A and B). These findings highlight the cell-context-dependent nature of K147R-mediated effects. To further evaluate the effect of HRAS lactylation on tumorigenesis, MDA-MB-468 cells expressing HRAS K117Q or K147Q mutants were subcutaneously injected into nude mice. Significantly, cells expressing HRAS K117Q or K147Q exhibited increased tumor growth compared to cells expressing HRAS-WT (Fig. 6C and D and Fig. S7C and D). Consistently, our stable site-specific HRAS lactylation system showed that K117La/K147La HRAS activated downstream signaling (p-ERK/p-MEK) and promoted anchorage-independent growth (Fig. S7E and F). Furthermore, genetic modulation of the lactylation machinery reinforced these observations: *TIP60* overexpression increased, whereas *TIP60* knockdown decreased, p-ERK/p-MEK signaling; SIRT2 showed the opposite effects (Fig. S7G and H). Importantly, SIRT2 knockdown, which enhances endogenous HRAS lactylation by blocking delactylation, significantly promoted tumor growth in vivo (Fig. S7I to K). These results provide convergent evidence that enhanced HRAS lactylation drives tumorigenesis in vivo. Clinical data analysis showed that the expression levels of mRNA and protein of HRAS and KRAS in breast cancer tissues were significantly higher than those in normal breast tissues (Fig. 6E and Fig. S7L). We also detected pan-RAS expression and pan-RAS lactylation in fresh breast cancer specimens and found that both were up-regulated in most paired breast cancer tissues compared with adjacent normal counterparts (Fig. 6F and Fig. S8A). In addition, we analyzed drug sensitivity data from the Genomics of Drug Sensitivity in Cancer (GDSC) database for HRAS-, KRAS-, and NRAS-mutant cell lines, showing that multiple compounds were effective against RAS-mutant cells, among which 3 drugs (selumetinib, PD0325901, and refametinib) were shared, all of which are clinically investigated MEK inhibitors (MEKis) (Fig. 6G and H and Fig. S8B). Further analysis confirmed that RAS mutations were more sensitive to these 3 drugs than WT-RAS (Fig. 6I to K). *LDHA* expression is significantly up-regulated in different cancers and positively correlated with lactate levels [30]. Correlation analysis showed a positive correlation between *LDHA* and both *HRAS* and *KRAS* expression (Fig. 6L and Fig. S8C). Consistently, *LDHA* expression significantly promoted, whereas the LDHA inhibitor (LDHi) sodium oxamate significantly inhibited, HRAS lactylation levels (Fig. 6M and Fig. S8D and E), indicating that LDHA inhibition may be a potential approach for treating patients with lactylation-mediated HRAS activation. We then evaluated the effects of the clinically approved drug stiripentol, recently identified as an LDHi, the MEKi selumetinib, or a combination on tumor cell viability, showing that each drug led to a significant reduction in tumor cell viability, and the combination was even more effective, with a significant synergistic effect (Fig. 6N and O and Fig. S8F to I). To establish causality, we used our stable site-specific lactylation system. In cells with HRAS-WT expression, both the LDHi and the MEKi individually suppressed cell growth, and their combination exhibited strong synergistic inhibition (Fig. S8J). In contrast, cells stably expressing site-specifically lactylated HRAS (K117La or K147La) were markedly less sensitive to LDHi and showed no significant synergy with MEKi, although they remained responsive to MEKi alone (Fig. S8J).

## Discussion

Our study reveals that lactylation of HRAS at K117 and K147, 2 mutation-prone residues in cancers, constitutively activates HRAS via disrupted GTP interaction, impaired GTPase activity, and enhanced protein stabilization. This discovery bridges PTMs and genetic mutations, redefining PTMs as dynamic mutation mimics in cancer biology (Fig. 6P).

### HRAS lactylation at mutation-prone hotspots promotes oncogenic functions by mimicking mutational activation and stabilization

TIP60, previously known for chromatin remodeling [31], is identified as a lactyltransferase targeting mutation-prone residues K117/K147, expanding its role as a metabolic modulator of HRAS. Conversely, SIRT2, a sirtuin deacylase, acts as the primary delactylase [20,21]. This reversible lactylation couples cellular lactate levels to HRAS activation, establishing it as a tightly regulated PTM. The TIP60–SIRT2 axis underscores lactylation as a reversible and tightly regulated PTM, dynamically coupling cellular metabolic states to HRAS activation.

Lactylation at K117 and K147 can neutralize the positively charged lysine side chains. For K117 lactylation, 2 distinct mechanisms may drive HRAS hyperactivity: (a) disruption of the hydrogen bond between K117 and GTP, impairing intrinsic GTPase activity, and (b) steric hindrance from the bulky lactyl group, which stabilizes GTP interaction while destabilizing catalytic interactions essential for GTP hydrolysis. In contrast, K147 lactylation mildly perturbs the backbone NH group’s interaction with GTP and neutralizes K147’s charge, altering the conformation of the GTP-binding pocket to favor HRAS-GTP accumulation. These modifications phenocopy mutations at the same sites, highlighting lactylation as a PTM-driven “pseudo-mutation” that bypasses genetic alterations to achieve constitutive HRAS activation.

Beyond GTPase regulation, K147 lactylation competitively antagonized ubiquitination at the same residue, shielding HRAS from LZTR1-mediated proteasomal degradation. This competition arises because lactylation and ubiquitination target the same lysine residue (K147), with lactylation physically occluding the ubiquitination site. Concurrently, USP32 enhances HRAS stability by removing residual ubiquitin chains, further amplifying oncogenic signaling. This interplay between lactylation and ubiquitination underscores a broader paradigm in tumorigenesis in which modification of mutation-prone residues disrupts protein homeostasis regulation.

Functionally, lactylated HRAS enhanced tumor cell viability, colony formation, and malignant transformation in vitro, recapitulating phenotypes driven by oncogenic mutations. Clinically, RAS lactylation was elevated in breast cancer tissues compared to adjacent normal tissues, highlighting its pathological relevance in the lactate-rich microenvironment associated with the Warburg effect. These findings position lactylation as a nonmutational driver of RAS hyperactivity, bridging metabolic rewiring to tumorigenesis.

Collectively, HRAS lactylation at K117/K147 mimics oncogenic mutations by disrupting GTP interaction, impairing GTPase activity, and stabilizing HRAS via competitive ubiquitination blockade. TIP60 and SIRT2 regulate this reversible PTM, linking lactate metabolism to HRAS activation.

### Our study indicates a potential therapeutic strategy for RAS lactylation-driven tumors

The Warburg effect creates a lactate-rich microenvironment that drives RAS lactylation [10–12]. Notably, our analysis of the GDSC database identified MEKis as the most effective agents against RAS-mutant cancers. We demonstrated that inhibiting lactate production via LDHAis reduced HRAS lactylation and synergized with MEKis to suppress tumor cells. This synergy highlights the therapeutic potential of dual metabolic and signaling pathway targeting, particularly in tumors with hyperactive WT-RAS. Given the mechanistic overlap between lactylation and mutations, inhibitors targeting RAS mutations may also benefit lactylation-driven cancers when combined with lactate-lowering therapies, offering a potential approach for WT-RAS-driven tumors.

### Our study redefines lactylation as a dynamic mutation mimic

We demonstrate that lactylation at mutation-prone residues mechanistically and functionally recapitulates genetic mutations. K117/147 lactylation is essentially identical to the mutation that replaces the unmodified K with an unnatural, noncoded amino acid (lactyl-K, i.e., lactylated K), as presented by the site-specific lactylation system. Thus, lactylation at mutation-prone residues is essentially an extension of mutation, serving as a dynamic mutation mimic. This paradigm may extend beyond lactylation, as the mechanistic principles suggest that other PTMs (e.g., acetylation and phosphorylation) at mutation hotspots may similarly operate as reversible “epi-mutations”. We thus introduce a paradigm-shifting concept that PTMs at mutation-prone residues function as dynamic mutation mimics where metabolic modification dynamically mimics genetic mutation effects, challenging the traditional dichotomy between genetic and epigenetic oncogenic drivers, explaining RAS hyperactivity in tumors lacking canonical mutations. Using the site-specific lactylation system to incorporate lactyl-K into HRAS that accurately preserves native steric and electrostatic properties while eliminating artifacts inherent to traditional mutagenesis, we demonstrate that lactylation alone recapitulates mutational effects, validating both the lactylation biology and conceptual advance.

This concept also indicates universal applicability across oncoproteins beyond RAS. Proteins with mutation hotspots [e.g., TP53 and EGF receptor (EGFR)] may similarly acquire gain-of-function phenotypes via PTMs that mimic or modify critical residues. The evolutionary conservation of K117 and K147 across the RAS superfamily further implies that lactylation-driven activation could be a conserved regulatory mechanism, warranting investigation in other GTPases.

This study has several limitations. First, our site-specific genetic code expansion system enables precise lactyl-lysine incorporation into HRAS, but parameters for in vivo application, including lactyl-lysine concentration and administration route, have not yet been optimized, precluding xenograft studies with fully lactylated HRAS. Second, K-to-R mutations are unsuitable as negative controls because K117R and K147R are themselves gain-of-function oncogenic mutations. For long-term studies, we therefore validated K-to-Q substitutions as the most appropriate surrogates, as glutamine’s polar side-chain (-NH₂) better mimics lactyl-lysine (-CH₃CHOH) than other substitutions. Third, for endogenous detection of RAS lactylation, prior immunoprecipitation with isoform-specific antibodies remains essential even when using our validated site-specific antibodies, due to high sequence conservation surrounding K117 and K147 across the RAS superfamily (>150 members). Fourth, while our mechanistic studies focused primarily on HRAS, the conceptual framework may extend to other RAS isoforms, given the conserved lactylation and regulatory mechanisms observed in KRAS and NRAS. Despite these technical constraints, our conclusions remain robust, supported by convergent validation through site-specific lactylation, targeted mutagenesis, and MD simulations.

## Conclusions

Our study reveals that HRAS lactylation at mutation-prone K117/K147 mimics oncogenic mutations, driving constitutive activation, malignant transformation, and tumorigenesis via disrupted GTP interaction, impaired GTPase activity, and enhanced protein stabilization. The TIP60–SIRT2 axis regulates this lactate-driven PTM. Our study also indicates that lactylation-mediated HRAS activation may be treated individually by inhibiting lactylation (e.g., LDHA blockade) or synergistically with mutation-targeted therapies (e.g., MEKis). By employing a site-specific lactylation system, we further redefine lactylation as a dynamic mutation mimic, revealing a conserved regulatory paradigm applicable to other oncoproteins, thus offering novel strategies to target PTM-driven pseudo-mutations.

## Ethical Approval

The study protocol was approved by the ethics committee of the Zhejiang University First Affiliated Hospital (approval number: 1045). Written informed consent was obtained from each patient to allow the use of his/her samples and data in research. The animal study was carried out in compliance with the guidance of the Animal Care Committee of Zhejiang University (approval number: AIRB-2025-0233).

## Data Availability

TCGA data were obtained from the TCGA-BRCA project (project ID: TCGA-BRCA; https://portal.gdc.cancer.gov/projects/TCGA-BRCA). METABRIC data (Molecular Taxonomy of Breast Cancer International Consortium) were accessed via cBioPortal (study ID: brca_metabric; https://www.cbioportal.org/study/summary?id=brca_metabric). Information on RAS mutations was obtained from the COSMIC (version 101; http://cancer.sanger.ac.uk/cancergenome/projects/cosmic). RAS interaction information was obtained from the Interaction Database (version 4.4; https://thebiogrid.org/). Proteomics data were obtained from the CPTAC-BRCA project (project ID: CPTAC-BRCA; https://cptac-data-portal.georgetown.edu/) and the ProteomeXchange Consortium via the PRIDE repository (PXD005692, PXD008841, and PXD011385). MS data of HRAS lactylation (OEP00005911) and ubiquitination (OEP00005922) have been deposited into the NODE database (The National Omics Data Encyclopedia). Other data generated during this study are included in this published article.
