# Supplementary material for: Lactylation of Mutation-Prone RAS Residues Mimics Oncogenic Mutations and Drives Constitutive Activation
Source: Cancer Commun (Lond). 2026 Aug 26;46:0047. doi: 10.34133/cancomm.0047 (PMC13507527; doi:10.34133/cancomm.0047)
Supplement: Supplementary file 1 [file cancomm.0047.f1.docx]

**Supplementary Materials**

**Lactylation of Mutation-prone RAS Residues Mimics Oncogenic Mutations and drives Constitutive Activation**

Ruocen Liao^1^^,2,†^, Chenglong Ma^1,†^, Xingyu Chen^3,†^, Longchang Bai^1,†^, Qianhua Cao^3^, Yu Zhang^1^, Chenfang Dong^1,2,*^

^1^Department of Surgical Oncology (Breast Center) and Department of Pathology and Pathophysiology, Key Laboratory of Cancer Prevention and Intervention, Ministry of Education, The Second Affiliated Hospital, Zhejiang University School of Medicine, Hangzhou, Zhejiang, P. R. China.

^2^Department of Breast Surgery, First Affiliated Hospital, Zhejiang University School of Medicine, Hangzhou, Zhejiang, P. R. China.

^3^Department of Respiratory and Critical Care Medicine, Key Laboratory of Respiratory Disease of Ningbo, The First Affiliated Hospital of Ningbo University, Ningbo, Zhejiang, P. R. China.

^†^Ruocen Liao, Chenglong Ma, Xingyu Chen, and Longchang Bai contributed equally to this study.

^*^Corresponding author:

Chenfang Dong, [chenfangdong@zju.edu.cn](mailto:chenfangdong@zju.edu.cn).

**Supplementary Figure S1. RAS was lactylated at the mutation-prone hotspots K117/K147, and its lactylation was mediated byTIP60 and SIRT2, related to Figure 1.**

**(A-B)** RAS expression in ER-positive and ER-negative breast cancer tissues was analyzed using the indicated datasets.

**(C-D)** Box plots show *RAS* expression across different histological grades of breast cancer in the METABRIC dataset.

**(E-G)** Kaplan–Meier survival analyses were performed using KM plotter ([https://kmplot.com](https://kmplot.com/)). Patients from the aggregate dataset (E) and Tang's dataset (F, G) were stratified by the upper quartile of *RAS* expression, and relapse-free survival (RFS) was compared using the log-rank test.

**(H)** Lactate (left) and glucose (right) levels were analyzed in normal and tumor tissues from Tang's metabolomics dataset.

**(I)** Lactate levels in normal tissues, breast cancer tissues of different subtypes, and breast cancer cell lines were analyzed using the metabolomics datasets of Terunuma (left) and CCLE (right).

**(J-L)** HRAS-WT or an empty vector control was expressed in the indicated cells. HRAS lactylation was assessed following immunoprecipitation using a pan-Kla antibody.

**(M-N)** HEK293T cells were transfected with the indicated Flag-HRAS plasmids and cultured in media containing the indicated concentrations of glucose (M) or lactate (N) for 24 h, followed by immunoprecipitation. Kla levels of the immunoprecipitated proteins were analyzed by western blotting.

**(O)** Sequence logo showing the conserved G4 and G5 motifs of the RAS superfamily.

Abbreviations: ER, estrogen receptor; ER^+^, estrogen receptor positive; ER^−^, estrogen receptor negative; METABRIC, Molecular Taxonomy of Breast Cancer International Consortium; CCLE, Cancer Cell Line Encyclopedia; WT, wild-type; HRAS, Harvey rat sarcoma viral oncogene homolog; Kla, lysine lactylation; G4, guanine nucleotide-binding box 4; G5, guanine nucleotide-binding box 5; IP, immunoprecipitation.

**Supplementary Figure S2. RAS lactylation promotes its activity and oncogenic functions, related to Figure 1.**

(A) Specificity of the site-specific K117 (top) and K147 (bottom) lactylation antibodies was evaluated by dot blot analysis.

**(B-D)** HRAS-WT, the K117R mutant (B), the K147R mutant (C), or the K117R/K147R double mutant (D) was expressed in HEK293T cells, followed by immunoprecipitation to determine K117 and K147 lactylation.

**(E)** Sequence alignment of the NKCD and SAK motifs in HRAS and their flanking amino acid sequences across representative RAS family members.

**(F-H)** Endogenous NRAS, HRAS, and KRAS in Hs578T (F), MDA-MB-468 (G), and MDA-MB-231 (H) cells were immunoprecipitated with the indicated isoform-specific antibodies or control IgG. The IP fractions and whole-cell lysates (Input) were analyzed by immunoblotting (IB) with K117La‑ and K147La‑specific antibodies to detect lactylation, as well as with pan‑RAS and isoform‑specific antibodies to confirm precipitation and loading, as indicated.

**(I)** Cell lysates from the indicated cell lines were subjected to pan-Kla antibody-based enrichment of lactylated proteins, followed by immunoblotting with HRAS-, KRAS-, and NRAS-specific antibodies.

**(J)** HEK293T cells were transfected with HRAS and/or AARS1 expression plasmids, and HRAS lactylation was assessed using a pan-Kla antibody.

**(K)** HEK293T cells were co-transfected with the indicated HDAC1–3 expression constructs, and HRAS lactylation was assessed using a pan-Kla antibody.

Abbreviations: NKCD, asparagine-lysine-cysteine-aspartate motif; SAK, serine-alanine-lysine motif; HRAS, Harvey rat sarcoma viral oncogene homolog; NRAS, neuroblastoma RAS viral oncogene homolog; KRAS, Kirsten rat sarcoma viral oncogene homolog; human breast cancer cell line; K117, lysine 117; K147, lysine 147; K117R, lysine 117 to arginine substitution; K147R, lysine 147 to arginine substitution; AARS1, alanyl-tRNA synthetase 1; Kla, lysine lactylation; HDAC, histone deacetylase.

**Supplementary Figure S3. RAS lactylation promotes its activity and oncogenic functions, related to Figure 2.**

**(A)** Site-specific incorporation of Kla into Flag-KRAS-K117TAG or K147TAG in HEK293T cells was confirmed by western blotting.

**(B)** HEK293T cells expressing HRAS-WT, site-specifically lactylated HRAS, or the indicated HRAS mutants were subjected to GST-RBD pull-down assays to assess GTP-bound RAS activity.

**(C)** RBD-GFP was co-expressed with HRAS-WT, site-specifically lactylated HRAS, or the indicated HRAS mutants in HEK293T cells, followed by immunoprecipitation and western blot analysis.

**(D)** Protein levels of p-ERK, ERK, p-MEK, and MEK in HEK293T cells expressing HRAS-WT, site-specifically lactylated HRAS, or the indicated HRAS mutant were examined by western blotting.

**(E)** Protein levels of p-ERK, ERK, p-MEK, and MEK in HEK293T cells expressing WT- or lactylated KRAS in growth medium containing 1% or 10% FBS were analyzed by western blotting.

**(F)** Protein levels of p-ERK, ERK, p-MEK, and MEK in HEK293T cells expressing WT-KRAS or different KRAS mutants were examined by western blotting.

**(G)** KRAS lactylation at K117 and K147 was assessed by immunoprecipitation followed by immunoblotting with K117La‑ and K147La‑specific antibodies in HEK293T cells co‑transfected with KRAS and TIP60, or the indicated constructs (left panel), as well as in Hs578T cells co‑expressing KRAS and *TIP60* shRNA#1/2 (right panel).

**(H)** KRAS lactylation at K117 and K147 was assessed by immunoprecipitation followed by immunoblotting with K117La‑ and K147La‑specific antibodies in HEK293T cells co‑transfected with KRAS and SIRT2, or the indicated constructs (left panel), as well as in Hs578T cells co‑expressing KRAS and *SIRT2* shRNA#1/2 (right panel).

**(I)** NIH/3T3 cells expressing HRAS-WT or HRAS-K147Q were injected into the mammary fat pad of nude mice. Tumor growth was monitored every 4 days. **P* < 0.05, ***P* < 0.01, ****P* < 0.001 by Student’s t-test.

Abbreviations: Kla, lysine lactylation; TAG, amber stop codon; GFP, green fluorescent protein; Mb-Pyl, Methanococcoides burtonii pyrrolysyl; RS, aminoacyl-tRNA synthetase; tRNA, transfer ribonucleic acid; KRAS, Kirsten rat sarcoma viral oncogene homolog; WT, wild-type; p-ERK, phosphorylated extracellular signal-regulated kinase; ERK, extracellular signal-regulated kinase; p-MEK, phosphorylated mitogen-activated protein kinase kinase; MEK, mitogen-activated protein kinase kinase; FBS, fetal bovine serum; HRAS, Harvey rat sarcoma viral oncogene homolog; K147Q, lysine 147 to glutamine substitution.

**Supplementary Figure S4. K117 lactylation promotes accumulation of GTP-bound active RAS, related to Figure 3.**

**(A)** The GTP-binding activity of purified HRAS-WT, HRAS mutants, or site-specifically lactylated HRAS-K117La/K147La was determined by GTP pull-down assay.

**(B)** The nucleotide exchange rate was assessed by incubating GDP-loaded HRAS-WT, HRAS-K117Q, and site-specifically lactylated HRAS-K117La with excess mant-GTP, and fluorescence changes were monitored over time.

**(C)** Superposition of the crystal structures of WT-RAS (PDB: 4Q21; tan) with K117R (PDB: 2QUZ; sky blue) or K117N (PDB: 8EIE; pink). An enlarged view of the GDP/GTP‑binding pocket is shown on the right.

**(D)** Superposition of the WT-RAS crystal structure (PDB: 4Q21; tan) and modeled HRAS-WT (sky blue). An enlarged view of the GDP/GTP‑binding pocket is shown on the right.

**(E-G)** Structural superposition of modeled HRAS-WT and K117Q (E), K117R (F), or K117N (G) mutants (left/top) and representative snapshots from MD simulations (right/bottom). An enlarged view of GTP is shown on the right.

**(H-J)** Overlay (left) or distribution (right) for GTP RMSD plots of MD trajectories in HRAS-WT, K117Q (H), K117R (I), and K117N (J) mutants.

**(K)** Overlay for protein backbone Cα RMSF profiles from MD trajectories in HRAS-WT, K117Q, K117R, and K117N mutants.

**(L-N)** Overlay (left) and distribution (right) of protein backbone Cα RMSF values comparing HRAS-WT with K117Q (L), K117R (M), and K117N (N), respectively. RMSF values were derived from MD simulations.

**(O)** Heatmap showing hydrogen-bond interactions between GTP and HRAS-WT, K117Q (left), K117R (middle), and K117N (right). Hydrogen-bond occupancy was defined as the proportion of simulation time during which each hydrogen bond was present.

Abbreviations: GST, glutathione S-transferase; RBD, RAS-binding domain; WT, wild-type; RAS, rat sarcoma viral oncogene homolog; PDB, Protein Data Bank; K117R, lysine 117 to arginine substitution; K117N, lysine 117 to asparagine substitution; K117Q, lysine 117 to glutamine substitution; GDP, guanosine diphosphate; GTP, guanosine triphosphate; HRAS, Harvey rat sarcoma viral oncogene homolog; MD, molecular dynamics; RMSD, root mean square deviation; RMSF, root mean square fluctuation; Cα, alpha carbon.

**Supplementary Figure S5. K147 lactylation promotes accumulation of active GTP-bound RAS, related to Figure 4.**

**(A)** The nucleotide exchange rate was measured by incubating GDP-loaded HRAS-WT, HRAS-K147Q, or HRAS-K147La (generated using the site-specific lactylation system) with excess mant-GTP, and monitoring fluorescence over time.

**(B-D)** Structural overlays of HRAS-WT with the K147Q (B), K147R (C), or K147N (D) mutant after modeling (left) or at representative snapshots from the MD trajectories (right). A magnified view of GTP is shown on the right.

**(E-G)** Overlay (left) and histogram (right) of GTP RMSD values from MD trajectories of HRAS-WT and the K147Q (E), K147R (F), or K147N (G) mutant.

**(H-K)** Overlay (left) and histogram (right; H–K) of protein backbone Cα RMSF values from MD trajectories of HRAS-WT and the K147Q (H), K147R (I), and K147N (J) mutants. **P* < 0.05, ***P* < 0.01, ****P* < 0.001 by Student’s t-test; ns, not significant.

**(L-M)** Overlay (L) and histogram (M) showing the distance between the backbone amide (NH) of residue 147 and the O6 atom of GTP in HRAS-WT, K147Q, K147R, and K147N during MD simulations.

**(N)** Heatmap for hydrogen-bonding interactions of GTP with HRAS-WT, K147Q (left), K147R (middle), and K147N (right) mutants. Occupancy was defined as the percentage (or fraction) of simulation time during which a given hydrogen bond was present.

Abbreviations: WT, wild-type; HRAS, Harvey rat sarcoma viral oncogene homolog; K147Q, lysine 147 to glutamine substitution; K147R, lysine 147 to arginine substitution; K147N, lysine 147 to asparagine substitution; GTP, guanosine triphosphate; MD, molecular dynamics; RMSD, root mean square deviation; RMSF, root mean square fluctuation; Cα, alpha carbon; NH, amide group; O6, oxygen 6 of guanine.

**Supplementary Figure S6. K147 lactylation stabilizes HRAS by competitively antagonizing protein ubiquitination, related to Figure 5.**

**(A)** Representative MS/MS spectra of HRAS peptides carrying K117 ubiquitination.

**(B)** HEK293T cells were co-transfected with the indicated plasmids, and HRAS ubiquitination was analyzed by ubiquitination assay.

**(C)** HEK293T cells were co-transfected with the indicated plasmids, and the ubiquitination of HRAS-WT, K147R, and K147La was assessed by ubiquitination assay.

**(D)** HEK293T cells expressing HRAS-WT, K147La, and/or LZTR1 were lysed and subjected to immunoprecipitation with anti-Flag M2 agarose, followed by western blot analysis of the indicated interactions. Quantification of the immunoblots is shown as bar graphs (mean ± SD).

**(E)** Partial list of HRAS-interacting proteins identified by proximity labeling mass spectrometry (PL-MS).

**(F)** *USP32* expression in normal breast and breast tumor tissues was analyzed using the TCGA dataset.

**(G)** Kaplan-Meier survival analysis of breast cancer patients stratified by *USP32* expression (high vs. low, based on median) in an aggregated dataset. The *P* value was calculated using the log-rank test.

**(H)** Protein expression of HRAS and USP32 was analyzed in the Johansson dataset. Relative HRAS expression was plotted against USP32 expression.

**(I)** Protein expression of HRAS and USP32 was analyzed by western blotting in SUM159 cells stably expressing either the empty vector or USP32.

**(J)** For the CHX chase assay, SUM159 cells stably expressing either the empty vector or USP32 were treated with 25 μg/mL CHX for the indicated times, and HRAS protein levels were analyzed by western blotting.

Abbreviations: MS/MS, tandem mass spectrometry; HRAS, Harvey rat sarcoma viral oncogene homolog; K117, lysine 117; HEK293T, human embryonic kidney 293 T cells; WT, wild-type; K117R, lysine 117 to arginine substitution; K147R, lysine 147 to arginine substitution; CUL3, cullin 3; LZTR1, leucine-zipper-like transcriptional regulator 1; PL-MS, proximity labeling mass spectrometry; USP32, ubiquitin-specific protease 32; TCGA, The Cancer Genome Atlas; SUM159, human breast cancer cell line; CHX, cycloheximide.

**Supplementary Figure S7. RAS lactylation was tumor-associated and inhibition of RAS lactylation sensitizes cancer cells to drugs targeting RAS mutations, related to Figure 6.**

**(A)** Anchorage-independent growth was evaluated by soft-agar assay in Hs578T cells expressing HRAS-WT or the indicated HRAS mutants. Data are presented as percentages relative to the empty vector control (mean ± SD from three independent experiments). **P* < 0.05, ***P* < 0.01, ****P* < 0.001 by Student’s t-test.

**(B)** Mammosphere formation of Hs578T cells expressing HRAS-WT or the indicated HRAS mutants was assessed. Data are presented as percentages relative to the empty vector control (mean ± SD from three independent experiments). **P* < 0.05, ***P* < 0.01, ****P* < 0.001 by Student’s t-test.

**(C-D)** MDA-MB-468 cells expressing HRAS-WT, HRAS-K117Q (C), or HRAS-K147Q (D) were orthotopically injected into the mammary fat pads of nude mice. Tumor growth was monitored every four days.

**(E)** Protein levels of p-ERK, ERK, p-MEK, and MEK were analyzed by western blotting in MDA-MB-468 cells expressing WT or lactylated HRAS.

**(F)** Anchorage-independent growth was evaluated by soft-agar assay in MDA-MB-468 cells expressing HRAS-WT, K117La, or K147La. Data are presented as percentages relative to the empty vector control (mean ± SD from three independent experiments). **P* < 0.05, ***P* < 0.01, ****P* < 0.001 by Student’s t-test.

**(G)** Protein levels of p-ERK, ERK, p-MEK, and MEK were analyzed by western blotting in HEK293T cells with or without TIP60 overexpression (left) and Hs578T cells with or without shTIP60 expression (right).

**(H)** Protein levels of p-ERK, ERK, p-MEK, and MEK were analyzed by western blotting in HEK293T cells with or without SIRT2 overexpression (left) and Hs578T cells with or without shSIRT2 expression (right).

**(I-K)** Hs578T cells expressing vector or shSIRT2 were injected into the mammary fat pad of nude mice. Tumor volume and tumor weight were measured (I). Tumor growth was monitored every four days (J). HRAS lactylation in harvested tumors was analyzed using site-specific lactylation antibodies (K). Data are presented as mean ± SEM (n = 6 mice per group). **P* < 0.05, ***P* < 0.01, ****P* < 0.001 by Student’s t-test.

**(L)** *RAS* mRNA expression in normal and tumor tissues was analyzed using the TCGA dataset.

Abbreviations: HRAS, Harvey rat sarcoma viral oncogene homolog; K117La, lysine 117 lactylation; K117Q, lysine 117 to glutamine substitution; K147La, lysine 147 lactylation; K147Q, lysine 147 to glutamine substitution; KRAS, Kirsten rat sarcoma viral oncogene homolog; MEK, mitogen-activated protein kinase kinase; p-ERK, phosphorylated extracellular signal-regulated kinase; p-MEK, phosphorylated mitogen-activated protein kinase kinase; SD, standard deviation; SEM, standard error of the mean; shRNA, short hairpin RNA; TCGA, The Cancer Genome Atlas; WT, wild-type.

**Supplementary Figure S8. RAS lactylation was tumor-associated and inhibition of RAS lactylation sensitizes cancer cells to drugs targeting RAS mutations, related to Figure 6.**

**(A)** Paired human breast tumor (T) and adjacent normal (N) tissues were lysed and subjected to western blot analysis of pan-RAS expression. For detection of pan-RAS lactylation, tissue lysates were immunoprecipitated with a pan-RAS antibody, followed by western blotting using site-specific RAS lactylation antibodies.

**(B)** Volcano plots showing the association between KRAS or NRAS mutations and sensitivity to different drugs (green, increased sensitivity; red, decreased sensitivity).

**(C)** Correlation analysis between KRAS and LDHA protein expression in the CPTAC dataset. The relative level of KRAS was plotted against that of LDHA.

**(D)** HEK293T cells were transfected with Flag-HRAS and/or LDHA expression plasmids, followed by immunoprecipitation. Kla levels of the immunoprecipitated HRAS were analyzed by western blotting, and quantification of the immunoblots is shown as bar graphs (mean ± SD).

**(E)** MDA-MB-231 cells expressing HRAS-WT were treated with or without sodium oxamate (20 mmol/L) for 24 h, followed by immunoprecipitation. Kla levels of the immunoprecipitated HRAS were analyzed by western blotting, and quantification of the immunoblots is shown as bar graphs (mean ± SD).

**(F)** Hs578T cells were treated with increasing concentrations of stiripentol (LDHA inhibitor) and selumetinib (MEK inhibitor) alone or in combination for 48 h. Dose–response matrices were generated based on cell viability measurements, and synergy scores were calculated using the ZIP (Zero Interaction Potency) model. .

**(G)** MDA-MB-468 cells were treated with increasing concentrations of stiripentol (LDHA inhibitor) and selumetinib (MEK inhibitor) alone or in combination for 48 h. Drug synergy was evaluated based on cell viability, and synergy scores were calculated using the ZIP (Zero Interaction Potency) model. The ZIP synergy score is shown in the matrix**.**

**(H)** MDA-MB-468 cells were treated with increasing concentrations of stiripentol (LDHA inhibitor) and selumetinib (MEK inhibitor) alone or in combination for 48 h. Dose–response matrices were generated based on cell viability measurements, and synergy scores were calculated using the ZIP (Zero Interaction Potency) model.

**(I)** Cell viability was measured after treatment of the indicated cells with selumetinib (0.08 mmol/L) and/or stiripentol (0.08 mmol/L) for the indicated times. Data are presented as mean ± SD from three independent experiments. **P* < 0.05, ***P* < 0.01, ****P* < 0.001 by Student’s t-test.

**(J)** MDA-MB-468 cells stably expressing HRAS-WT, K117La, or K147La were treated with selumetinib (0.08 mmol/L) and/or stiripentol (0.08 mmol/L) for the indicated times, followed by measurement of cell viability. Data are presented as percentages relative to the control cells (mean ± SD from three independent experiments).

Abbreviations: HRAS, Harvey rat sarcoma viral oncogene homolog; KRAS, Kirsten rat sarcoma viral oncogene homolog; NRAS, neuroblastoma RAS viral oncogene homolog; WT, wild-type; SD, standard deviation; K117Q, lysine 117 to glutamine substitution; K147Q, lysine 147 to glutamine substitution; K117N, lysine 117 to asparagine substitution; K117R, lysine 117 to arginine substitution; K147N, lysine 147 to asparagine substitution; K147R, lysine 147 to arginine substitution; TCGA, The Cancer Genome Atlas; LDHA, lactate dehydrogenase A; Kla, lysine lactylation; IP, immunoprecipitation.
